# Impact of aortic angulation on outcomes in transcatheter aortic valve replacement with balloon-expandable and self-expanding valves: a systematic review and meta-analysis

**DOI:** 10.1007/s12928-025-01169-8

**Published:** 2025-07-18

**Authors:** Basma Badrawy Khalefa, Ahmed Reda Gonnah, Mazen Negmeldin Aly Yassin, Hossam Fayed, Moumen Arnaout, Mohamed Karam Allah Elkholy, Mohamed Ramadan, Abdelrahman Mohammed Elettreby, Ali Dway, Hatem Eldeeb, Abdullah Saeed Abujabal, David Hesketh Roberts

**Affiliations:** 1 Faculty of Medicine, Ain Shams University, Cairo, Egypt; 2 Department of Medicine, Imperial College Healthcare NHS Trust, London, UK; 3 Faculty of Medicine, Cairo University, Cairo, Egypt; 4 Faculty of Medicine, Al-Azhar University, Cairo, Egypt; 5 Faculty of Medicine, Aleppo University, Aleppo, Syria; 6 Faculty of Pharmacy, Zagazig University, Zagazig, Egypt; 7 Faculty of Medicine, Suez University, Suez, Egypt; 8 Faculty of Medicine, Mansoura University, Mansoura, Egypt; 9 Faculty of Medicine, Al-Andalus University for Medical Sciences, Tartus, Syria; 10 Lancashire Cardiac Centre, Blackpool, UK; 11 School of Medicine, University of Liverpool, Liverpool, UK

**Keywords:** Aortic angulation, TAVR, BEV, SEV

## Abstract

High aortic angulation (AA) can pose significant challenges during TAVR. This meta-analysis determines the impact of a horizontal aorta on TAVR outcomes with balloon-expandable (BEV) and self-expanding valves (SEVs). A comprehensive search was conducted from inception to June 2024. Thirteen observational studies assessing the impact of aortic angulation in patients undergoing TAVR replacement were included. The pooled results indicated that short-term mortality was significantly lower in patients with a non-horizontal aorta (RR = 0.76; 95% CI 0.62–0.95, *P* = 0.01). Subgroup analysis displayed that BEVs had a lower short-term mortality with a horizontal aorta than SEVs. The incidence of stroke was not significantly affected by aortic angulation with either valve type. However, the overall risk of permanent pacemaker implantation was lower in patients with a non-horizontal aorta for both valve types. Paravalvular regurgitation was also reduced in the non-horizontal aorta group, with no difference between aortic angulations in patients with BEVs. In contrast, SEVs favored a non-horizontal aorta in reducing paravalvular leak (RR = 0.66; 95% CI 0.46–0.94, *P* = 0.02). Both BEVs and SEVs show better results in patients with lower aortic root angulation. The increased incidence of conduction abnormalities and PPI rates with both valve types in patients with horizontal aorta is a concern. BEVs could be potentially used preferentially to SEVs in patients with a horizontal aorta as there was no difference between horizontal and non-horizontal aorta groups regarding short-term mortality, moderate-to-severe paravalvular leak, and need for a second valve.

**Supplementary Information:**

The online version contains supplementary material available at 10.1007/s12928-025-01169-8.

## Introduction

Transcatheter aortic valve replacement (TAVR) is a safe procedure that is increasingly used to treat patients with aortic stenosis, utilizing both self-expanding valves (SEVs) and balloon-expandable valves (BEVs) [1–5]. High aortic angulation (AA), known as a horizontal aorta, can pose significant challenges during TAVR [6–8]. Asymmetric valve deployment may adversely affect the success of the procedure and lead to post procedural complications. Sherif et al*.* evaluated the correlation between higher AA and lower procedural success in patients treated with the first-generation SEVs, and also showed an increased rate of paravalvular aortic regurgitation [9]. A subsequent study reported that increased AA adversely affected acute procedural success after the first-generation SEVs, but not after the first-generation BEVs [10]. However, another study conducted on the first-generation SEVs reported no association between AA and procedural success or clinical outcomes [11]. Subsequent studies using newer generations of valves reported that increasing AA does not affect the outcomes in patients who undergo TAVR with SEVs or BEVs [12–16]. However, other studies reported that new-generation SEVs, such as Evolut R/PRO and Portico, may be not appropriate in patients with a horizontal aorta [17–19].

This systematic review and meta-analysis is to determine the influence of SEVs and BEVs on clinical outcomes in patients with a horizontal aorta (HA).

## Methods

Our methodology and findings were conducted according to the Cochrane Handbook for Systematic Reviews and reported according to the Preferred Reporting Items for Systematic Reviews and Meta-Analyses (PRISMA) [20, 21]. Transparency was ensured by registering the protocol of our study in PROSPERO (CRD42024549955).

### Search strategy

We performed an extensive search across various databases, such as PubMed, Embase, Web of Science, Scopus, and the Cochrane Library, from inception to 5 June 2024. We utilized the following strategy: ((Transcatheter aortic valve replacement) OR (Transcatheter aortic valve implantation) OR (TAVI) OR (TAVR)) AND ((Angulation*) OR (Horizontal)).

### Study selection and eligibility criteria

The articles found during the systematic search were added to the EndNote Reference Library [22], where duplicates were identified and eliminated. After excluding the duplicates, initial screening of the titles and abstracts via Rayyan website was performed, followed by detailed review of chosen study texts [23]. We included observational studies or clinical trials that compared the outcomes between patients with horizontal and non-horizontal aorta undergoing TAVR with any valve type. Only studies with a cut-off angle between horizontal and Non-horizontal aorta between 48 and 52 degrees were included. We excluded case reports, case series studies, reviews, abstracts, animal, non-English studies, and studies with a cut-off angle higher than 52 or lower than 48 degrees.

### Data extraction

Extraction forms were constructed on Google Spread Sheets. We extracted the following information for each study:

1. Summary of the included studies including study design, follow-up duration, aortic angle cut of point, country, inclusion criteria, and exclusion criteria; details are provided in Table 1.
2. Baseline characteristics including age, sample size, gender, number of patients with diabetes mellitus, hypertension, conduction disturbances, stroke or transient ischemic attack, prior myocardial infarction (MI), and Society of Thoracic Surgeons (STS) score. Details are provided in Table 2.
3. Primary outcomes including death, stroke, MI, major bleeding, and secondary outcomes including length of hospital stay, aortic dissection, duration of the procedure, need of second valve, cardiac tamponade, aortic regurgitation, permanent pacemaker implantation, procedure success, and paravalvular leak.
4. Procedural characteristics including access, pre-dilation, post-dilation, valve type, and valve size. Details are provided in Supplementary Table 1.

**Table 1:** Summary of characteristics of included studies

Study ID	Study design	Follow-up duration		Aortic angle cut of point		Country	Type of used valves	Inclusion criteria	Exclusion criteria	Study conclusion
		Non-horizontal group	Horizontal group	Non-horizontal group	Horizontal group					
Abramowitz et al. 2016	Retrospective cohort	30 days, 6 months	30 days, 6 months	AA < 48°	AA ≥ 48°	USA	BEV, SEV	Patients with severe symptomatic aortic stenosis who underwent TAVR and had contrast CT available for AA evaluation	Patients with aortic angulation (AA) > 70 and patients who did not have concurrent contrast CT scans available for analysis of AA	Increased aortic root angulation adversely affects the procedural success following SE TAVR but not balloon-expandable (BE) TAVR. Therefore, BE valves may be preferred in patients with high AA when considering TAVR
Aktan et al. 2023	Retrospective cohort	In hospital, 30 days, 1 year	In hospital, 30 days, 1 year	AA ≤ 48°	AA > 48°	Turkey	SEV	Patients who underwent transfemoral-approach TAVR with the Evolut R valve between August 2015 and August 2022 and had their AA evaluated by MSCT	Patients with a history of pacemaker implantation or surgical aortic valve replacement, balloon-expandable (BE) TAVR, valve-in valve procedure, bicuspid aortic valve, no evaluable MSCT prior to TAVR, no transfemoral access, and valve-in-valve TAVR	Increased AA does not have a significant impact on intraprocedural and periprocedural complications in patients with new-generation self-expandable valves implanted
Aslan et al. 2022	Retrospective cohort	30 days	30 days	AA ≤ 48°	AA > 48°	Turkey	SEV	Patients who underwent a TAVR with a self-expanding Portico TAVR system from March 2017 to October 2021	Bicuspid valve (*n* = 5), previously implanted surgical bioprosthetic valves (*n* = 1), and inability to access all medical records (*n* = 1)	Increased AA is associated with higher rates of PAR and valve malposition, with the self-expanding portico valve, despite comparable device success and early outcome rates
Barki et al. 2023	Retrospective cohort	90 day	90 day	AA < 48°	AA ≥ 48°	Italy	SEV	Patients with symptomatic severe aortic valve stenosis undergoing TAVR with self-expanding neo and neo2 valves	Pure aortic regurgitation (AR) patients, valve-in-valve procedures, and all approach besides the transfemoral and the trans subclavian access	HA represents a frequent anatomical feature in patients with severe aortic valve stenosis undergoing TAVR and emerged as a relevant risk factor for developing ≥ moderate PVL
Bob-Manuel et al. 2019	Retrospective cohort	30 days, 6 months, 1 year	30 days, 6 months, 1 year	AA < 49°	AA > 49°	USA	BEV, SEV	Patients who underwent TAVR from May 2014 to June 2017 and had severe symptomatic aortic stenosis, and had MSCT angiography studies available for aortic angulation evaluation	Patients were excluded if they did not have MSCT images for pre-TAVR planning, used an obsolete method of measuring aortic angulation, or received first-generation valves	The study concluded that increasing aortic angulation does not significantly affect short- or long-term outcomes in patients who underwent TAVR with new-generation balloon-expandable or self-expandable valves
D’Ancona et al. 2019	Prospective cohort	30 days	30 days	AA < 50°	AA ≥ 50°	Germany	SEV	Patients undergoing TAVR with Evolut R prosthesis for whom complete pre- operative and perioperative data were available, including CT images with AA calculation, and periprocedural intra-hospital information	Patients with bicuspid AV and AV annular dimensions below or above the recommended limits proposed by the manufacturing company, as well as patients with previous AVR or TAVR	AA did not affect procedural outcomes and valve performance of the Evolut R prosthesis
Eckel et al. 2024	Retrospective cohort	30 days	30 days	AA < 51.7°	AA ≥ 51.7°	Germany	BEV, SEV	Patients with symptomatic severe AS, that were treated with TF‐TAVR between January 2017 and January 2023 using the Sapien3 Ultra prosthesis or Neo2 prosthesis	Patients with prior surgical aortic valve replacement and prior transcatheter aortic valve replacement	Transfemoral TAVR in patients with severe aortic stenosis and HA, using the balloon-expandable Sapien3 Ultra and self‐expanding ACURATE Neo2 prosthesis, is feasible and safe. Therefore, valve selection between these platforms should be made irrespective of the aortic angle
Gallo et al. 2021	Retrospective cohort	30 days	30 days	AA < 49°	AA ≥ 49°	Multi-center	SEV	Patients who underwent transfemoral TAVR for severe aortic stenosis of the native AV with either Evolut R/PRO or ACURATE neo devices from September 2014 to April 2020	Patients undergoing TAVR for pure aortic regurgitation, surgical prosthesis degeneration, or from non-transfemoral access and patients undergoing Evolut R 34 mm implantation	Horizontal aorta, as defined by an AA ≥ 49°, is a common feature among transcatheter aortic valve replacement candidates and predicts device failure of the Evolut R/PRO valves, but not of the ACURATE neo valve. AA may be an effect modifier of the association between self-expanding valve type and device success
Medranda et al. 2021	Retrospective cohort	30 days	30 days	AA < 48°	AA ≥ 48°	USA	BEV, SEV	Patients who had contrast CT available for AA evaluation pre-TAVR and those who underwent transfemoral TAVR using a contemporary THV, defined as either the third-generation BE SAPIEN 3 or the third-generation SE CoreValve Evolut PRO/PRO +	Patients were excluded if they did not have evaluable pre-TAVR CT, underwent non-TF TAVR, or underwent valve-in-valve TAVR	AA no longer plays a role with new-generation BE or SE THVs in contemporary TAVR practice. AA of 48 did not affect procedural success or in-hospital outcomes and should no longer be a consideration when determining THV selection
Popma et al. 2016	Retrospective cohort	30 days	30 days	AA < 50.6°	AA ≥ 50.6°	USA	SEV	Patients with New York Heart Association (NYHA) functional class II or greater symptoms were eligible for inclusion	Exclusion criteria included an aortic annular diameter < 18 mm or > 29 mm. Patients were treated with a 23-mm, 26-mm, 29-mm, or 31-mm CoreValve device	The degree of Aortic valve angulation does not affect early clinical outcomes of self-expanding transcatheter aortic valve replacement
Rashid et al. 2017	Retrospective cohort	30 days	30 days	AA < 48°	AA ≥ 48°	Australia	MEV	All patients with severe AS that underwent TAVR with the Lotus Valve system between April 2012 and November 2016	NA	AA did not affect device success or clinical outcome with the Lotus Valve system. The Lotus’ unique design features may have mitigated the impact of AA by improving the accuracy and ease of valve positioning
Stefano et al. 2019	Retrospective Cohort	30 days	30 days	AA < 48°	AA ≥ 48°	Italy	BEV, MEV, SEV	Patients with severe symptomatic AS, who underwent transfemoral TAVR between March 2012 and December 2017 with the second-generation aortic bioprostheses	Patients with a bicuspid aortic valve, primary aortic regurgitation, subaortic obstructive membrane, previous cardiac surgery, those undergoing a valve-in-valve procedure or non-transfemoral access or with failure of femoral access and those without good preprocedural CT scan	The degree of aortic angulation (AA) does not significantly impact outcomes after TAVR with BE valves
Veulemans et al. 2020	Retrospective cohort	30 days	30 days	AA < 51°	AA ≥ 51°	Germany	SEV	Severe symptomatic aortic stenosis (AS) Transfemoral TAVR Self-expandable device (Corevalve Evolut R or Pro)	Bicuspid aortic valve Prior aortic valve replacement Insufficient MSCT data	An aortic root angulation above 51° is associated with an increased rate of stroke, major vascular complications, and 30-day mortality following transfemoral TAVR with self-expandable new-generation devices

*USA* United States of America, *TAVR* transcatheter aortic valve replacement, *CT* computed tomography, *AA* aortic angulation, *SE* self -expandable, *MSCT* multi-slice computed tomography, *PAR* paravalvular aortic regurgitation, *AV* aortic valve, *AVR* aortic valve replacement, *THV* transcatheter heart valve, *TF* transfemoral, *BE* balloon-expandable, *AS* aortic stenosis, *MEV* mechanically expandable valves

**Table 2:** Baseline characteristics of included patients

Study ID	Valve used	Sample size		Age (mean ± SD)		Male		Diabetes mellitus		Hypertension	
		Non-HA	HA	Non-HA	HA	Non-HA	HA	Non-HA	HA	Non-HA	HA
Abramowitz et al. 2016 (SEV)	SEV and BEV	299	283	81.6 ± 9.1	82.4 ± 7.7	183 (61.2)	171 (60.4)	97 (32.4)	91 (32.2)	267 (89.3)	259 (91.5)
Aktan et al. 2023	SEV	149	115	79.0 ± 6.5	78.8 ± 6.3	62 (41.6)	57 (49.6)	38 (25.5)	27 (23.5)	92 (61.7)	60 (52.2)
Aslan et al. 2022	SEV	64	57	80.4 ± 6.2	80.0 ± 6.1	19 (29.7)	25 (43.9)	27 (42.2)	24 (42.1)	39 (60.9)	39 (68.4)
Barki et al. 2023	SEV	493	407	NA	NA	NA	NA	NA	NA	NA	NA
Bob-Manuel et al. 2019 (SEV)	SEV	28	24	80.3 ± 7.0	77.8 ± 9.5	12 (42.9)	11 (45.8)	NA	NA	NA	NA
Bob-Manuel et al. 2019 (BEV)	BEV	64	63	79.5 ± 8.25	78.6 ± 9.1	36 (56.25)	38 (60)	NA	NA	NA	NA
D’Ancona et al. 2019	SEV	70	76	81.6 ± 6.2	82.3 ± 6.3	23(32.9)	35(45.7)	NA	NA	NA	NA
Eckel et al. 2024	SEV and BEV	841	741	NA	NA	NA	NA	NA	NA	NA	NA
Gallo et al. 2021	SEV	2039	1823	81.5 ± 6.2	82.3 ± 5.6	770 (37.8)	641 (35.2)	599 (29.4)	527 (29)	1747 (85.8)	1586 (87.3)
Medranda et al. 2021 (SEV)	SEV	229	111	78.0 ± 9.6	80.2 ± 8.3	105 (45.9)	50 (45)	89 (38.9)	35 (61.5)	202 (88.2)	97 (87.4)
Medranda et al. 2021 (BEV)	BEV	293	208	77.9 ± 9.7	80.7 ± 8.6	168 (57.3)	134 (64.4)	108 (36.9)	75 (36.1)	255 (87.0)	191 (91.8)
Popma et al. 2016	SEV	2503	1075	82.8 ± 7.9	84.6 ± 7.3	1384 (55.3)	564 (52.5)	954 (38.1)	382 (35.5)	2330 (93.1)	1006 (93.6)
Rashid et al. 2017	MEV	78	87	83.5 ± 5.7	83.8 ± 5.7	37 (47)	42 (48)	20 (26)	15 (17)	60 (77)	64 (74)
Stefano et al. 2019	BEV, SEV and MEV	317	230	80.6 ± 7.9	83.1 ± 5.9	150 (47.3)	104 (45.2)	72 (22.7)	50 (21.8)	249 (78.5)	197 (86)
Veulemans et al. 2020	SEV	225	241	81.7 ± 5.4	82.0 ± 5.7	87 (38.6)	104 (46.2)	65 (28.9)	79 (32.8)	205 (91.1)	226 (93.8)

Study ID	Chronic kidney disease		Peripheral artery disease		Stroke or TIA		Prior MI		Prior PCI		Prior CABG	
	Non-HA	HA	Non-HA	HA	Non-HA	HA	Non-HA	HA	Non-HA	HA	Non-HA	HA
Abramowitz et al. 2016 (SEV)	45 (15.1)	43 (15.2)	93 (31.1)	93 (32.9)	64 (21.4)	64 (22.6)	NA	NA	NA	NA	75 (25.1)	69 (24.4)
Aktan et al. 2023	32 (27.8)	45 (30.2)	3 (2.0)	4 (3.5)	3 (2.0)	1 (0.9)	NA	NA	44 (29.5)	41 (35.7)	16 (10.7)	14 (12.2)
Aslan et al. 2022	21 (32.8)	13 (22.8)	14 (21.9)	14 (24.6)	1 (1.6)	4 (7.0)	NA	NA	NA	NA	11 (17.2)	16 (28.1)
Barki et al. 2023	NA	NA	NA	NA	NA	NA	NA	NA	NA	NA	NA	NA
Bob-Manuel et al. 2019 (SEV)	NA	NA	10 (35.7)	2 (8.3)	3 (10.7)	4 (16.7)	NA	NA	NA	NA	NA	NA
Bob-Manuel et al. 2019 (BEV)	NA	NA	16 (25)	14 (22.5)	7 (11)	12 (19)	NA	NA	NA	NA	NA	NA
D’Ancona et al. 2019	NA	NA	NA	NA	NA	NA	NA	NA	NA	NA	NA	NA
Eckel et al. 2024	NA	NA	NA	NA	NA	NA	NA	NA	NA	NA	NA	NA
Gallo et al. 2021	NA	NA	287 (14.2)	208 (1809)	214 (10.6)	195 (10.8)	375 (18.6)	287 (15.8)	577 (28.6)	476 (26.3)	280 (13.9)	157 (8.7)
Medranda et al. 2021 (SEV)	NA	NA	32 (14)	15 (13.5)	17 (7.4)	8 (7.2)	42 (18.3)	9 (8.1)	54 (23.6)	19 (17.1)	43 (18.8)	13 (11.7)
Medranda et al. 2021 (BEV)	NA	NA	65 (22.2)	32 (15.4)	23 (7.8)	18 (8.7)	49 (16.7)	22 (10.6)	94 (32.1)	58 (27.9)	60 (20.5)	36 (17.3)
Popma et al. 2016	NA	NA	1171 (46.8)	455 (42.3)	596 (23.8)	261 (24.3)	NA	NA	1009 (40.3)	371 (34.5)	919 (36.7)	357 (33.2)
Rashid et al. 2017	26 (29)	20 (23)	NA	NA	9 (11)	8 (9)	8 (10)	7 (8)	14 (18)	14 (16)	NA	NA
Stefano et al. 2019	164 (51.7)	119 (51.7)	57 (18)	37 (16.1)	29 (9.1)	40 (17.5)	49 (15.5)	35 (15.3)	93 (29.3)	59 (25.8)	55 (17.4)	14 (6.1)
Veulemans et al. 2020	NA	NA	55 (24.4)	55 (22.8)	NA	NA	NA	NA	85 (37.8)	86 (35.7)	19 (8.4)	25 (10.4)

Study ID	STS score (mean ± SD)		LVEF, (%)	
	Non-HA	HA	Non-HA	HA
Abramowitz et al. 2016 (SEV)	7.7 ± 4.6	7.8 ± 4.8	56.1 ± 15.1	58.0 ± 13.8
Aktan et al. 2023	8.7 ± 2.7	8.5 ± 2.8	50.1 ± 11.9	51.7 ± 11.3
Aslan et al. 2022	6.8 ± 3.18	5.4 ± 4	54.8 ± 8.3	53.5 ± 10.6
Barki et al. 2023	NA	NA	NA	NA
Bob-Manuel et al. 2019 (SEV)	6.02 ± 3.3	6.0 ± 3.4	58.25 ± 11.4	48.75 ± 15.2
Bob-Manuel et al. 2019 (BEV)	6.99 ± 5.6	7.15 ± 4.04	56 ± 12.1	55.68 ± 11.85
D’Ancona et al. 2019	4.43 ± 2.37	4.2 ± 2.5	55.3 ± 13.0	52.0 ± 13.4
Eckel et al. 2024	NA	NA	NA	NA
Gallo et al. 2021	4.7 ± 3.6	4.7 ± 3.8	57 ± 10.5	57.6 ± 10.6
Medranda et al. 2021 (SEV)	4.7 ± 3.8	5.2 ± 4.2	55.2 ± 13.5	55.7 ± 12.8
Medranda et al. 2021 (BEV)	5.4 ± 3.9	5.2 ± 3.2	52.3 ± 14.3	52.5 ± 15.2
Popma et al. 2016	8.7 ± 4.7	8.5 ± 4.3	NA	NA
Rashid et al. 2017	4.4 ± 2.6	4.0 ± 2.3	58.1 ± 14.5	58.0 1 ± 2.0
Stefano et al. 2019	5.9 ± 4.9	5.5 ± 4.2	54.5 ± 11.4	54.1 ± 11.3
Veulemans et al. 2020	5.7 ± 4.9	6.7 ± 5.8	56.2 ± 13.4	54.0 ± 13.8

### Risk-of-bias assessment (ROB)

The quality assessment of the included studies was performed using the Newcastle–Ottawa scale (NOS) [24]. NOS covers the following three domains: (1) selection of study groups; (2) comparability of groups; and (3) ascertainment of exposure and outcomes. NOS’s total score of 7 to 9 indicates a low risk of bias, 4 to 6 indicates a moderate risk, and 0 to 3 indicates a high risk of bias. Detailed ROB is provided in Supplementary Table 2.

### Data analysis

We conducted our analysis using R studio software [25]. We estimated the risk ratio (RR) for dichotomous outcomes and the mean difference (MD) for continuous outcomes. Given the substantial heterogeneity among the included studies, we preferred to use the random-effects model. We used the 95% confidence intervals (CI) for all outcomes. A *P* value < 0.05 indicated statistical significance. Chi-square *P* value < 0.10 indicated significant heterogeneity among the included studies.

### Definitions

All outcomes were defined according to VARC criteria [26, 27]. Details about specific criteria used in each study are provided in Supplementary Table 3. Additionally, definitions of the aortic angle in each study are provided in Supplementary Table 4.

## Results

### Literature search

**Fig. 1:**
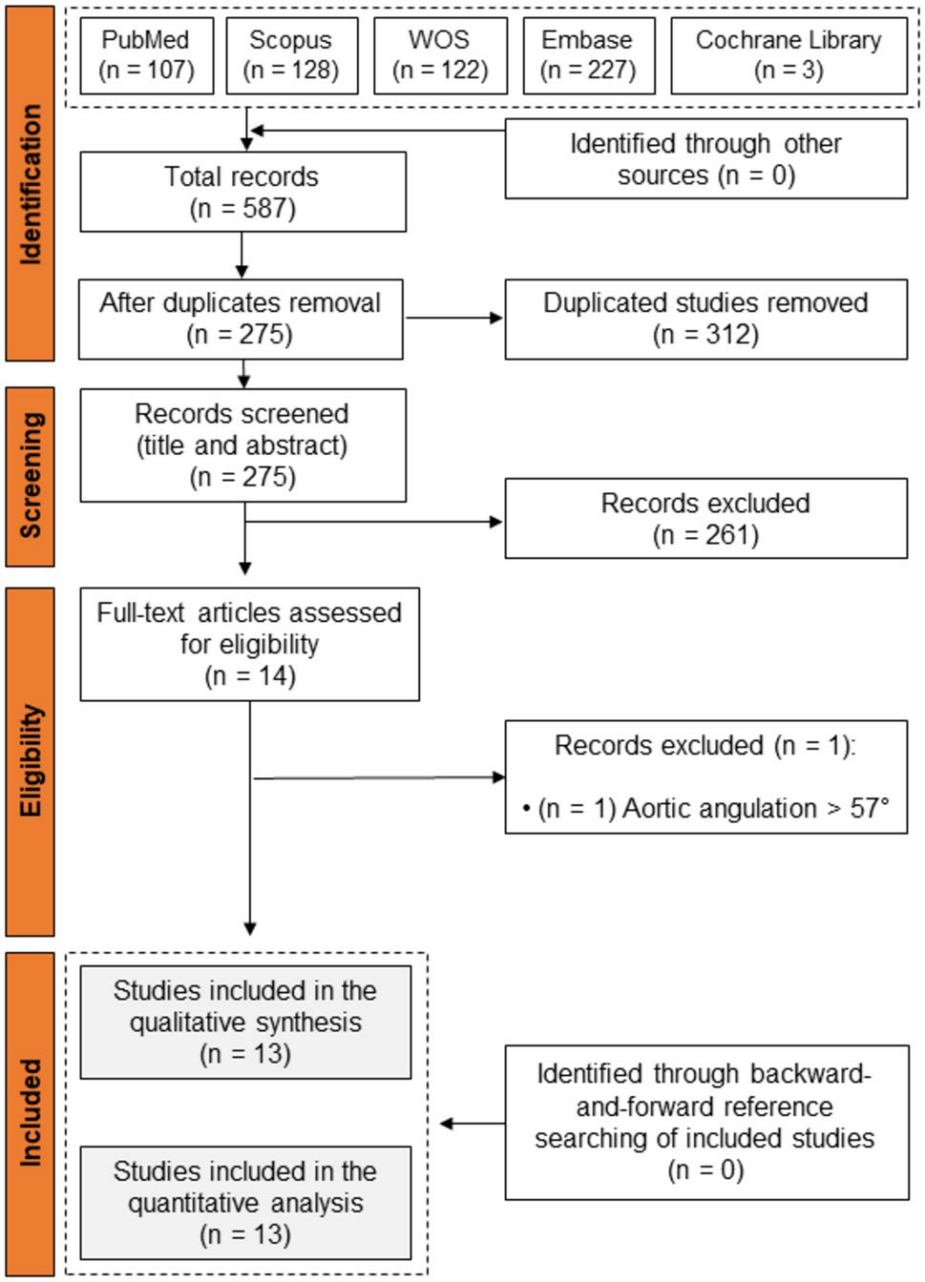
PRISMA flow diagram

Our search strategy produced a total number of 587 records, which were reduced to 275 articles after removal of duplicated studies. Following full-text screening, 13 studies satisfied our inclusion criteria and were identified for inclusion in our study [10–17, 19, 28–31]. Details are provided in PRISMA flowchart, Fig. 1.

### Characteristics of included studies

Thirteen studies were included in our systematic review and meta-analysis, with a total of 5541 patients in horizontal aorta group and 7692 patients in the non-horizontal aorta group. All of the included studies were observational studies, and most of the studies were conducted in the United States (40%) and Turkey (20%). The mean age of patients in the horizontal aorta group was 83.2 ± 7.5 and 82.1 ± 8.1 in the non-horizontal aorta group.

### Risk-of-bias assessment

Newcastle–Ottawa scale determined that all the included studies pose low risk of bias. Detailed ROB is provided in Supplementary Table 2.

### Outcomes of TAVR in horizontal versus non-horizontal aorta

#### Short-term (30-day) mortality

**Fig. 2:**
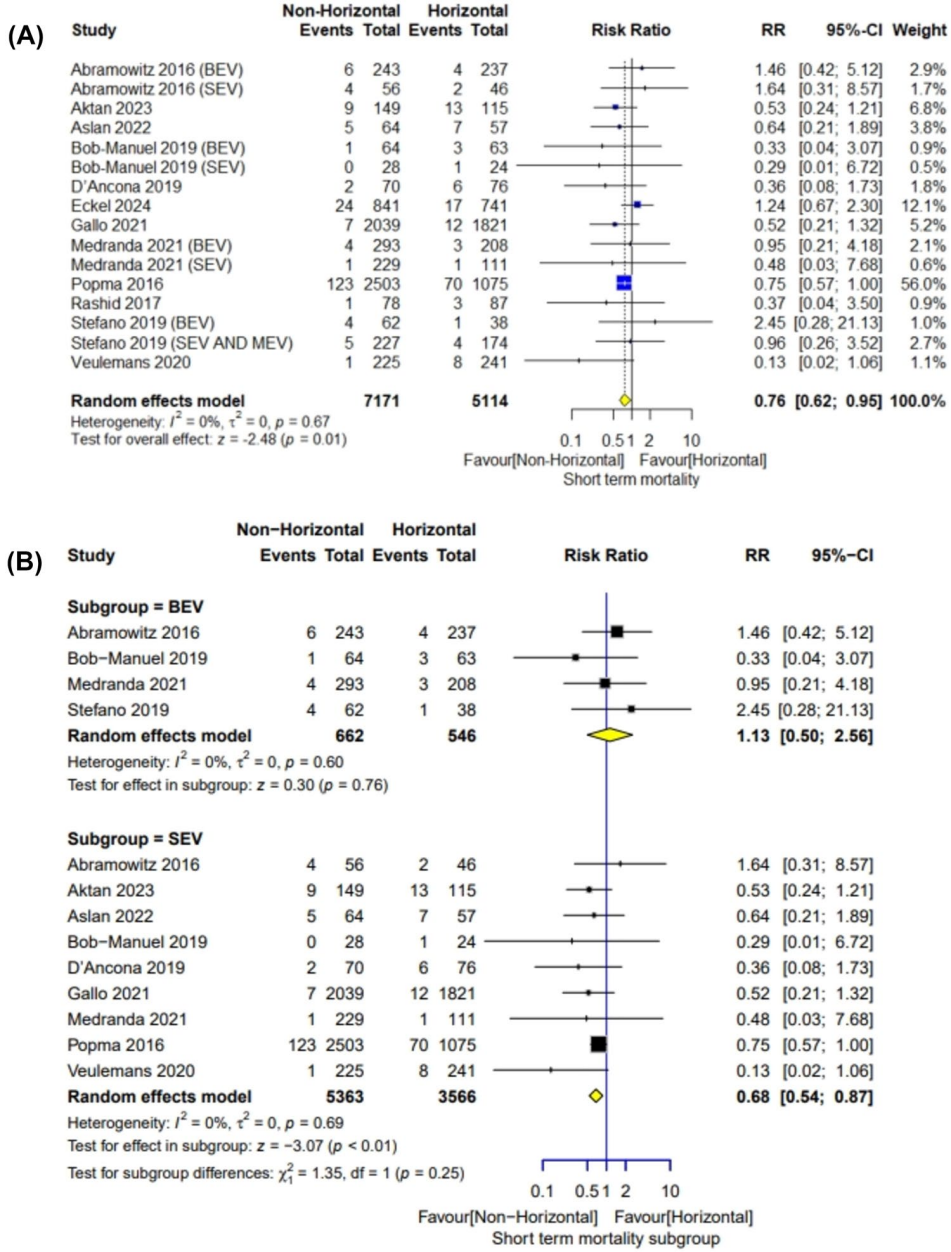
Short-term (30-day) mortality forestplot

The meta-analysis included 12,285 patients. The overall risk ratio favored the non-horizontal aorta group (RR = 0.76; 95% CI [0.62–0.95], *P* = 0.01). Pooled studies demonstrated no heterogeneity (*I*^2^ = 0%; *P* = 0.67). Analysis based on the type of valve used favored the non-horizontal aorta in the SEV subgroup (RR = 0.68; 95% CI [0.54–0.87], *P* < 0.01) (*I*^2^ = 0%; *P* = 0.69). The overall risk ratio did not favor the horizontal or non-horizontal aorta in the BEV subgroup (RR = 1.13; 95% CI [0.50–2.56], *P* = 0.76) (*I*^2^ = 0%; *P* = 0.60) (Fig. 2). On performing subgroup analysis based on valve generation, we did not find any difference between non-horizontal and horizontal groups on using Corevalve (RR = 0.77; 95% CI [0.58–1.02], *P* = 0.07) (*I*^2^ = 0%; *P* = 0.36), but the use of Evolut R favored the non-horizontal group (RR = 0.48; 95% CI [0.24–0.97], *P* = 0.04) (*I*^2^ = 0%; *P* = 0.86) (Fig. S1).

#### One-year mortality

**Fig. 3:**
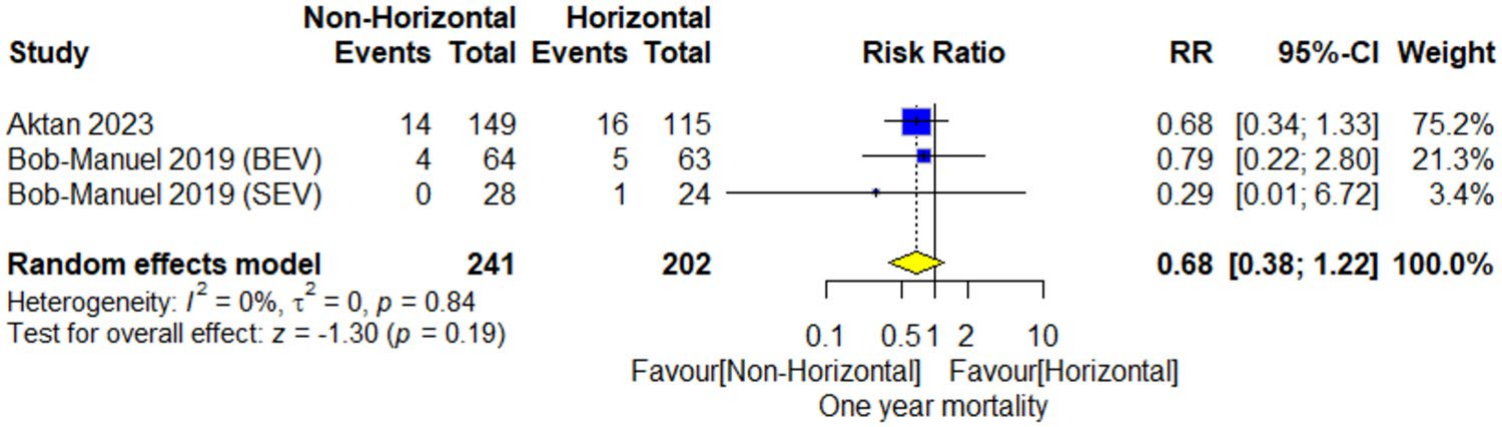
One-year mortality forestplot

The meta-analysis included 443 patients. The overall risk ratio did not favor either of both groups (RR = 0.68; 95% CI [0.38–1.22], *P* = 0.19). Pooled studies demonstrated no heterogeneity (*I*^2^ = 0%; *P* = 0.84) Fig. 3.

#### Stroke

**Fig. 4:**
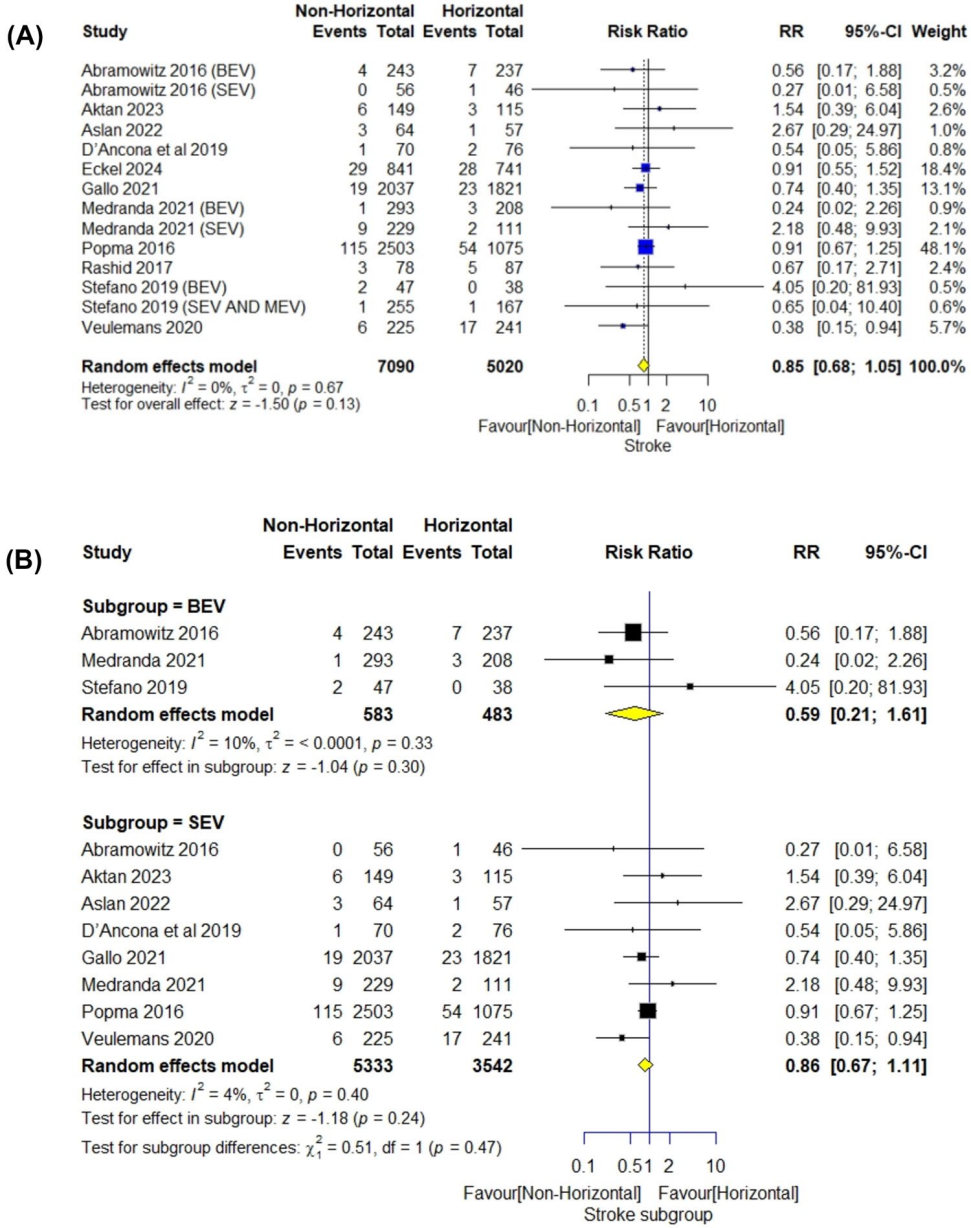
Stroke forestplot

The meta-analysis included 12,110 patients. The overall risk ratio did not favor any aortic angulation (RR = 0.85; 95% CI [0.68–1.05], *P* = 0.13). Pooled studies were homogenous (*I*^2^ = 0%; *P* = 0.67). There was no difference between the horizontal and non-horizontal groups with BEVs (RR = 0.59; 95% CI [0.21–1.61], *P* = 0.30) (*I*^2^ = 10%; *P* = 0.33) and with SEVs (RR = 0.86; 95% CI [0.67–1.11], *P* = 0.24) (*I*^2^ = 4%; *P* = 0.40) (Fig. 4). On subgroup analysis according to valve generation, we did not find any difference between both anatomical groups when using Corevalve (RR = 0.9; 95% CI [0.66–1.24], *P* = 0.53) (*I*^2^ = 0%; *P* = 0.46), or Evolut R (RR = 1.19; 95% CI [0.36–3.89], *P* = 0.77) (*I*^2^ = 0%; *P* = 0.46) (Fig. S2).

#### Myocardial infarction (MI)

**Fig. 5:**
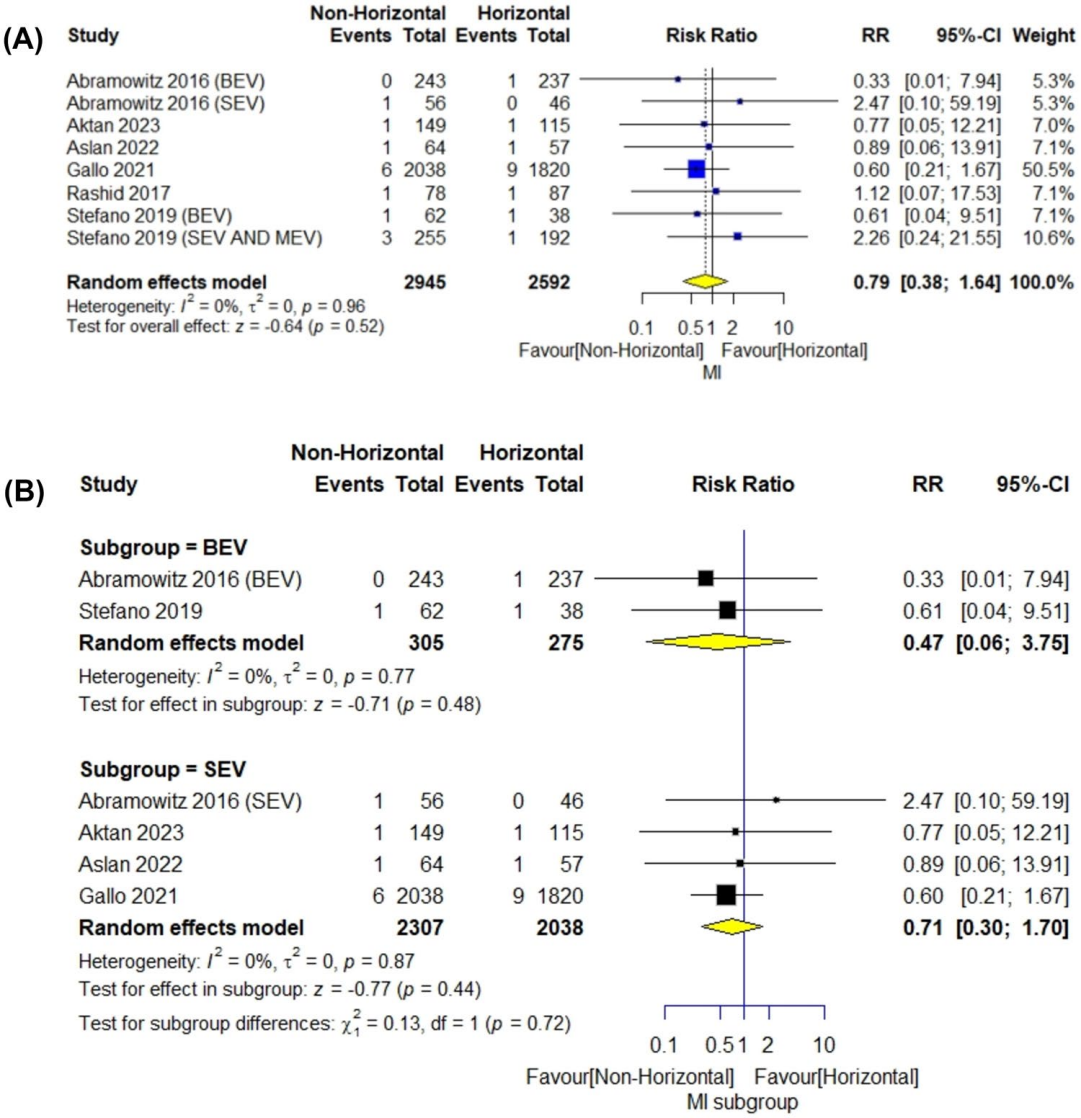
Myocardial infarction forestplot

The meta-analysis included 5537 patients. The overall frequency of MI was similar with both a horizontal and non-horizontal aorta (RR = 0.79; 95% CI [0.38–1.64], *P* = 0.52), with no statistical difference between horizontal and non-horizontal aorta with using BEVs or SEVs (RR = 0.47; 95% CI [0.06–3.75], *P* = 0.48) (*I*^2^ = 0%; *P* = 0.77) and (RR = 0.71; 95% CI [0.30–1.70], *P* = 0.44) (*I*^2^ = 0%; *P* = 0.87), respectively. Pooled studies were homogenous (*I*^2^ = 0%; *P* = 0.96) (Fig. 5).

#### Major or life-threatening bleeding

**Fig. 6:**
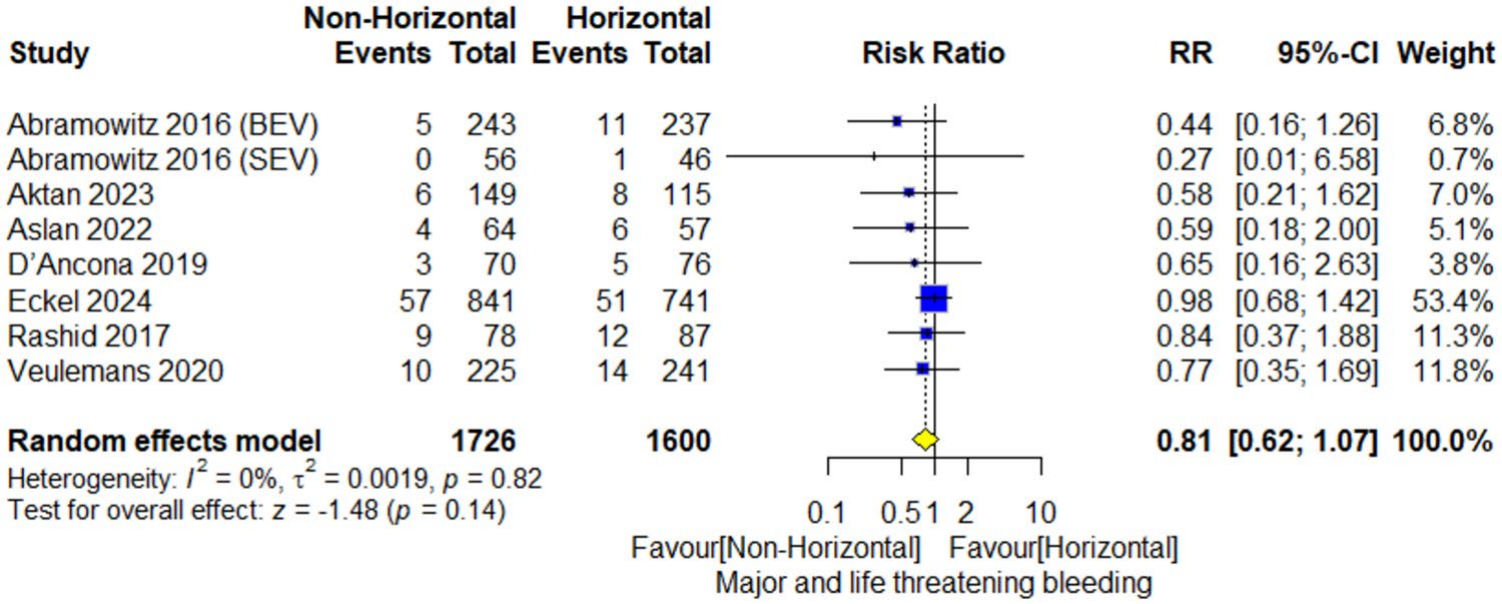
Major or life-threatening bleeding forestplot

The meta-analysis included 3,326 patients. The overall risk ratio did not favor a non-horizontal aorta (RR = 0.81; 95% CI [0.62–1.07], *P* = 0.14). Pooled studies were homogenous (*I*^2^ = 0%; *P* = 0.82) (Fig. 6).

#### New permanent pacemaker implantation (PPI)

The meta-analysis included 12,156 patients. The overall risk of PPI rates was lower with a non-horizontal aorta (RR = 0.87; 95% CI [0.79–0.94], *P* < 0.01). PPI rates were also lower in the non-horizontal group with both BEVs and SEVs (RR = 0.67; 95% CI [0.47–0.96], *P* = 0.03) (*I*^2^ = 8%; *P* = 0.35) and (RR = 0.88; 95% CI [0.80–0.97], *P* < 0.01) (I^2^ = 0%; *P* = 0.92), respectively (Fig. S3). Pooled studies did not display heterogeneity (*I*^2^ = 0%; *P* = 0.89). On subgroup analysis, we found no difference between both anatomical groups when using both Corevalve (RR = 0.89; 95% CI [0.72–1.11], *P* = 0.31) (*I*^2^ = 14%; *P* = 0.28), and Evolut R (RR = 0.81; 95% CI [0.48–1.38], *P* = 0.45) (*I*^2^ = 0%; *P* = 0.74) (Fig. S4).

#### Moderate or severe aortic regurgitation (AR)

The meta-analysis included 8433 patients. The overall risk of AR did not favor either a horizontal or non-horizontal aorta (RR = 0.99; 95% CI [0.75–1.30], *P* = 0.93). Pooled studies showed non-significant heterogeneity (*I*^2^ = 21%; *P* = 0.26), and also there was no difference between horizontal and non-horizontal aorta with the use of BEVs or SEVs (RR = 1.12; 95% CI [0.44–2.85], *P* = 0.81) (I2 = 0%; *P* = 0.46) and (RR = 0.89; 95% CI [0.71–1.13], *P* = 0.34) (*I*^2^ = 0%; P = 0.47), respectively (Fig. S5).

#### Moderate or severe paravalvular leak (PVL)

The meta-analysis included 4096 patients. The overall risk of significant PVL favored the non-horizontal aorta group (RR = 0.70; 95% CI [0.55–0.90], *P* < 0.01). PVL was increased with a SEV in a horizontal aorta (RR = 0.66; 95% CI [0.46–0.94], *P* = 0.02) (*I*^2^ = 0%; *P* = 0.45), but not with a BEV in a horizontal aorta (RR = 0.92; 95% CI [0.27–3.14], *P* = 0.89) (*I*^2^ = 0%; *P* = 0.84). Pooled studies demonstrated no heterogeneity (*I*^2^ = 0%; *P* = 0.76) (Fig. S6).

#### Left bundle branch block (LBBB)

The meta-analysis included only two studies with a total of 3112 patients. The overall risk of new LBBB was less in the non-horizontal aorta group (RR = 0.80; 95% CI [0.68–0.94], *P* < 0.01). Pooled studies were homogenous (*I*^2^ = 0%; *P* = 0.41) (Fig. S7).

#### Major vascular complications

The meta-analysis included 6904 patients. The overall risk ratio did not favor any group (RR = 0.89; 95% CI [0.69–1.13], *P* = 0.33). Pooled studies demonstrated no heterogeneity (*I*^2^ = 0%; *P* = 0.65) (Fig. S8). When performing subgroup analysis based on valve generation, we did not find any difference between both anatomical groups on using Corevalve (RR = 0.8; 95% CI [0.62–1.03], *P* = 0.08) (*I*^2^ = 0%; *P* = 0.49), or Evolut R (RR = 0.7; 95% CI [0.34–1.46], *P* = 0.34) (*I*^2^ = 0%; *P* = 0.95) (Fig. S9).

#### Acute kidney injury (AKI)

The meta-analysis included 10,802 patients. The overall risk ratio did not favor any group (RR = 0.97; 95% CI [0.74–1.27], *P* = 0.82). It also did not favor any group with BEVs (RR = 1.76; 95% CI [0.62–4.97], *P* = 0.29) (*I*^2^ = 0%; *P* = 0.82) or SEVs (RR = 0.81; 95% CI [0.64–1.02], *P* = 0.07) (*I*^2^ = 0%; *P* = 0.56). Pooled studies displayed non-significant heterogeneity (*I*^2^ = 19%; *P* = 0.26) (Fig. S10). Subgroup analysis based on valve generation revealed no difference between both non-horizontal and horizontal groups with the use of Corevalve (RR = 0.96; 95% CI [0.78–1.18], *P* = 0.69) (*I*^2^ = 0%; *P* = 0.9), or Evolut R (RR = 0.83; 95% CI [0.31–2.18], *P* = 0.7) (*I*^2^ = 0%; *P* = 0.7) (Fig. S11).

#### Annular rupture

Only two studies with a total of 4309 patients reported the incidence of annular rupture. The overall risk ratio did not favor either of the two groups (RR = 0.47, [95% CI 0.13–1.74], *P* = 0.26). The pooled studies showed no heterogeneity (*I*^2^ = 0%, *P* = 0.67) (Fig. S12).

#### Valve embolization

The meta-analysis included 9908 patients. The overall risk ratio did not favor any anatomical group (RR = 0.82; 95% CI [0.60–1.13], *P* = 0.22). Also there was no difference between both anatomical groups with using BEVs (RR = 0.77; 95% CI [0.11–5.41], *P* = 0.79) (*I*^2^ = 0%; *P* = 0.82) or SEVs (RR = 0.85; 95% CI [0.60–1.19], *P* = 0.34) (*I*^2^ = 9%; *P* = 0.36). Pooled studies were homogenous (*I*^2^ = 0%; *P* = 0.78) (Fig. S13).

#### Cardiac tamponade

The meta-analysis included 1,598 patients. The overall risk ratio did not favor either aortic groups (RR = 1.10; 95% CI [0.47–2.59], *P* = 0.83). Pooled studies were homogenous (*I*^2^ = 0%; *P* = 0.90) (Fig. S14).

#### Coronary obstruction

The meta-analysis included 5273 patients. The overall risk ratio did not favor either aortic groups (RR = 0.94; 95% CI [0.37–2.37], *P* = 0.90). Pooled studies displayed no heterogeneity (*I*^2^ = 0%; *P* = 0.88) (Fig. S15).

#### Need for second valve

The meta-analysis included 5508 patients. The overall risk ratio significantly favored the non-horizontal aorta group (RR = 0.62; 95% CI [0.42–0.91], *P* = 0.02) and significantly favored the non-horizontal aorta group in the SEV subgroup (RR = 0.50; 95% CI [0.26–0.93], *P* = 0.03) (*I*^2^ = 26%; *P* = 0.26), but did not favor either aortic angulation groups in the BEV subgroup (RR = 0.89; 95% CI [0.26–3.04], *P* = 0.85) (*I*^2^ = 0%; *P* = 0.77). Pooled studies did not demonstrate heterogeneity (*I*^2^ = 0%; *P* = 0.60) (Fig. S16).

#### Total contrast used, mL

The meta-analysis included 8478 patients. The volume of contrast used was not affected by the degree of aortic angulation (MD = − 2.12; 95% CI [− 6.37 to 2.13], *P* = 0.33) and the overall effect size did not favor any group with using either BEV or SEV (MD = 2.23; 95% CI [− 2.77 to 7.24], *P* = 0.38) (*I*^2^ = 0%; *P* = 0.56) and (MD = − 5.48; 95% CI [− 12.06 to 1.09], *P* = 0.10) (*I*^2^ = 46%; *P* = 0.09), respectively. Pooled studies revealed significant heterogeneity (*I*^2^ = 49%; *P* = 0.02) (Fig. S17).

#### Total fluoroscopy time (minutes)

The meta-analysis included 6444 patients. The overall mean difference significantly favored the non-horizontal aorta group (MD = − 1.02; 95% CI [− 1.98 to − 0.07], *P* = 0.04). The overall effect size did not favor either group with using the BEV or the SEV subgroups (MD = − 0.12; 95% CI [− 1.15 to 0.92], *P* = 0.82) (*I*^2^ = 0%; *P* = 0.50) and (MD = − 1.54; 95% CI [− 3.75 to 0.66], *P* = 0.17) (*I*^2^ = 72%; *P* < 0.01), respectively. The pooled studies showed significant heterogeneity (*I*^2^ = 58%; *P* = 0.01) (Fig. S18).

#### Length of hospital-stay (days)

The meta-analysis included 1717 patients. The overall mean difference did not favor either aortic angulation groups (MD = − 0.67; 95% CI [− 1.58 to 0.24], *P* = 0.15). The pooled studies showed significant heterogeneity (*I*^2^ = 78%; *P* < 0.01) (Fig. S19).

## Discussion

All studies included in our analysis defined aortic root angulation > 48° as a minimum cut-off point, which was determined by a multidetector contrast computed tomographic (MDCT) imaging of the aorto-valvular complex (a prerequisite for case planning) as a horizontal aorta [10, 17, 19, 28]. Case planning using MDCT has become an integral part of contemporary TAVR practice, and has a profound impact on the reduction of residual AR, due to appropriate bioprosthetic sizing [32, 33]. Additionally, it allows the identification of unfavorable calcium morphology within the aortic annulus and left-ventricular outflow tract, which may enhance the selection of the transcatheter heart valve [34, 35]. Pre-procedural sizing of the aortic root and identification of the origin of the coronary arteries is essential and has been associated with reduced occurrence of coronary occlusion during the procedure [36, 37].

The meta-analysis of TAVR outcomes in patients with horizontal versus non-horizontal aortas showed several significant findings. In terms of short-term mortality, the non-horizontal aorta group was significantly favored with a risk ratio (RR) of 0.76. The analysis of 1-year mortality and stroke incidence did not yield significant differences between the two groups. For PPI, the non-horizontal aorta group was again significantly favored with an RR of 0.87. The rate of myocardial infarction, moderate or severe aortic regurgitation showed no significant differences. However, for moderate or severe paravalvular leak, the non-horizontal aorta group was significantly favored with an RR of 0.70.

For SEVs, on pooling of the nine studies, patients with a non-horizontal aorta demonstrated a significantly lower short-term mortality rate compared to those with a horizontal aorta, with a risk ratio (RR) of 0.68. Additionally, the incidence of new PPI was significantly reduced, with an RR of 0.88. The non-horizontal aorta group also had fewer cases of moderate or severe paravalvular regurgitation, as indicated by an RR of 0.66, and a lower need for a second valve, with an RR of 0.5. However, all other outcomes including but not limited to stroke, myocardial infarction, and moderate or severe aortic regurgitation did not show significant differences between the non-horizontal and horizontal aorta groups for SEVs. For BEVs, on pooling of the four studies, the non-horizontal aorta group showed a significant advantage only in the incidence of new PPI, with an RR of 0.67. All other outcomes, such as short-term mortality, stroke, and moderate or severe paravalvular leak (PVL), did not exhibit significant differences between the non-horizontal and horizontal aorta groups.

There has been a variability in the generations of SEVs and BEVs used in this meta-analysis. While our meta-analysis includes data from both early and newer generation SEVs, it is important to highlight that recent advancements in device engineering, such as in the Evolut PRO + and ACURATE Neo2, have improved outcomes in complex anatomies, including horizontal aortas. Studies like Barki et al. have shown promising performance of the ACURATE Neo2 in such anatomies, suggesting that certain newer-generation SEVs may mitigate the risks previously associated with horizontal aorta. Future research should aim to stratify outcomes by device generation to further clarify these effects [38]. Some individual studies evaluating SEVs in patients with horizontal aorta displayed reduced procedural success [10, 17] and increased procedural complications, including PVL, valve malposition [19], stroke, major vascular complications, and 30-day mortality [28]. Other studies evaluating both SEVs and BEVs identified that the degree of aortic valve angulation did not affect the procedural success, short- or long-term clinical outcomes [10, 17], concluding that increased aortic root angulation should no longer be a consideration when determining transcatheter heart valve selection. Manoharan et al., identified that newer generations of SEVs, such as Evolur R improve the ability to treat patients with more complex aortic anatomy [39]. Barki et al.’s multicenter cohort study (900 patients, 107 with Neo2 in horizontal aorta) found that the Neo2 achieves comparable device success and a significantly lower rate of ≥ moderate PVL (5% vs. 15% with Neo) in horizontal anatomy, with no correlation between paravalvular leak and aortic angulation for Neo2 [38]. However, Gallo et al. have identified horizontal aorta, defined by an AA ≥ 49°, as a predictor of device failure of the Evolut R/PRO valves [17]. Similarly, Aslan et al. displayed an increased risk of paravalvular aortic regurgitation and valve malposition with the Portico prosthesis [19]. This variability warrants us to interpret the results of the studies that has shown worse outcomes with SEVs in horizontal aorta neutrally [17, 32–35, 39].

Non-horizontal aorta offers more stable valve anchoring and alignment. The better outcomes associated with non-horizontal aorta in patients receiving SEV are likely attributed to this more favorable anatomical alignment, which facilitates optimal deployment and reduces procedural complications [8]. The lower rates of PPM implantation with SEVs in non-horizontal aorta are potentially due to the more predictable expansion dynamics [8]. Moreover, SEVs have a longer stent frame, which is associated with increased paravalvular aortic regurgitation, post-dilatation, conduction abnormalities, and need for second valve [10]. Interestingly, although the incidence of valve embolization was not significantly different between horizontal and non-horizontal aorta groups, the need for a second valve was notably higher in horizontal aortas treated with SEVs. This discrepancy may be attributed to difficulties in optimal initial deployment due to misalignment or under-expansion, particularly in steep angulation where coaxiality is compromised. Such suboptimal positioning can lead to significant PVL or incomplete expansion, necessitating valve-in-valve implantation to correct the issue. Furthermore, certain SEVs with supra-annular configurations may be more susceptible to asymmetric deployment, increasing the risk of prosthesis dysfunction even without complete embolization. Sherif et al. evaluated 50 patients who underwent self-expanding CoreValve TAVR [9]. They assessed aortic angulation using left ventriculography in a right anterior oblique projection of 30° during preparation of the patients for the procedure. They found a greater chance of significant paravalvular aortic regurgitation with a greater aortic angulation. The main limitations of this study were the relatively small number of patients, the evaluation of only SEVs, and the method of aortic angulation assessment, which did not include CT, which is considered a more accurate modality to assess the aortic valve and aortic root before TAVR [7]. Additionally, SEVs included in this analysis varied considerably in terms of design characteristics, including frame height, radial force, deployment mechanism, and recapturability. For example, the ACURATE Neo and Neo2 systems feature a top–down deployment from stabilizing arches, which may enhance coaxiality and procedural success in the setting of a horizontal aorta. Gallo et al. demonstrated this advantage in the HORSE registry, suggesting that the ACURATE Neo system may perform more favorably in anatomies with high angulation [17]. Conversely, supra-annular devices like CoreValve and Evolut R/PRO, while offering hemodynamic advantages, have shown increased risk of device malposition in these anatomies. Therefore, while our meta-analysis broadly reports the pooled effects of SEVs, valve-specific behaviors and performance must be interpreted with caution, as they likely influence outcomes in horizontal aorta more than in non-horizontal configurations.

For BEVs, the significant finding of lower PPI rates in non-horizontal aorta suggests that the alignment of the aorta may influence the precision of balloon expansion and valve placement, reducing mechanical interference with the conduction system [8]. The overall comparable outcomes for other parameters such as short-term mortality and moderate-to-severe paravalvular leak indicate that BEVs performance is less influenced by the aortic angulation compared to SEVs. BEVs have a shorter frame stent which could be the potential reason behind successful deployment in any angulation [10]. Moreover, the shorter frame stent in BEVs makes it superior to SEVs in terms of conduction disturbances and new PPI, irrespective of the orientation [8, 10].

These findings are consistent with the previous studies that highlighted the challenges posed by horizontal aorta in TAVR procedures using SEVs. Studies have suggested that horizontal aorta can complicate valve deployment, leading to suboptimal outcomes [6, 9, 40]. Additionally, valve size and asymmetric calcification affect the repositioning maneuvers, which could explain the suboptimal outcomes in TAVR patients with increased aortic root angular undergoing SEV implantation [10]. Moreover, subsequent Valve Academic Research Consortium-2 (VARC-2) adverse events, indicated that a horizontal aorta, along with specific anatomic features, remains a crucial factor for TAVR related outcome with self-expanding devices [10, 17, 19, 28]. Our results corroborate these findings, especially in the context of SEVs, where the mechanical properties and deployment mechanisms appear more sensitive to aortic angulation [10, 17, 19, 28]. In contrast, the relative uniformity in outcomes for BEVs aligns with prior research, which indicated that the balloon-expandable mechanism might offer more consistent results irrespective of aortic orientation [8, 10–13, 15].

### Impact on clinical practice

In terms of clinical practice implications, the results suggest that patients with non-horizontal aorta benefit from TAVR with SEV or BEV. For patients with horizontal aorta, valve selection should be carefully considered, with BEV potentially offering more predictable outcomes. In cases of contraindications to BEVs, such as heavy left-ventricular outflow tract and sinotubular junction calcium, SEVs can be cautiously used in patients with high AA, however, with appropriate procedural planning, and guiding patients through the risks. These insights can guide preprocedural planning with MDCT, including detailed anatomical assessment and selection of the most appropriate valve type, based on accurate valve annulus sizing and aortic valve calcium score, to optimize patient outcomes, and particularly reduce the incidence of conduction abnormalities and pacemaker implantation, which was significant in patients with increased aortic angulation undergoing either SEV or BEV implantation. Although our analysis displays worse outcomes of SEV implantation in patients with horizontal aorta, appropriate new-generation SEV selection can potentially be associated with improved procedural and device implantation success. Further research is warranted to ascertain this finding.

### Strengths and limitations

The study's strengths include comprehensive inclusion of multiple studies, enhancing statistical power and reliability of the findings, and detailed comparison of a wide range of clinical outcomes. However, there are limitations, such as the limited number of studies for certain outcomes, patient populations, and procedural techniques, and not accounting for potential confounders like operator experience, patient baseline characteristics, different BEVs and SEVs types, as well as specific anatomical variations beyond aortic angulation. Moreover, the aortic root angulation cut-offs were variable in the studies included, and hence, the results of some studies have to be interpreted with caution.

## Conclusions

Both BEVs and SEVs have shown better results in TAVR patients who have a lower aortic root angulation. The increased incidence of conduction abnormalities with both valve types in patients with horizontal aorta, and PPI rates is a concern in this patient group. BEVs could be potentially used preferentially to SEVs in patients with a horizontal aorta as they are less affected by aortic angulation regarding the incidence of short-term mortality, moderate-to-severe paravalvular leak and need for second valve as opposed to SEVs.

## Supplementary Information

Below is the link to the electronic supplementary material.

Supplementary file1 (DOCX 5676 KB)

Supplementary file2 (DOCX 26 KB)

Supplementary file3 (DOCX 17 KB)

Supplementary file4 (DOCX 16 KB)

## Data Availability

All data generated or analyzed during this study are included in this published article.
