# Supplementary material for: Impact of aortic angulation on outcomes in transcatheter aortic valve replacement with balloon-expandable and self-expanding valves: a systematic review and meta-analysis
Source: Cardiovasc Interv Ther. 2025 Jul 18;40(4):746–66. doi: 10.1007/s12928-025-01169-8 (PMC12431928; doi:10.1007/s12928-025-01169-8)
Supplement: Supplementary file 1 — Supplementary file1 (DOCX 5676 KB) [file 12928_2025_1169_MOESM1_ESM.docx]

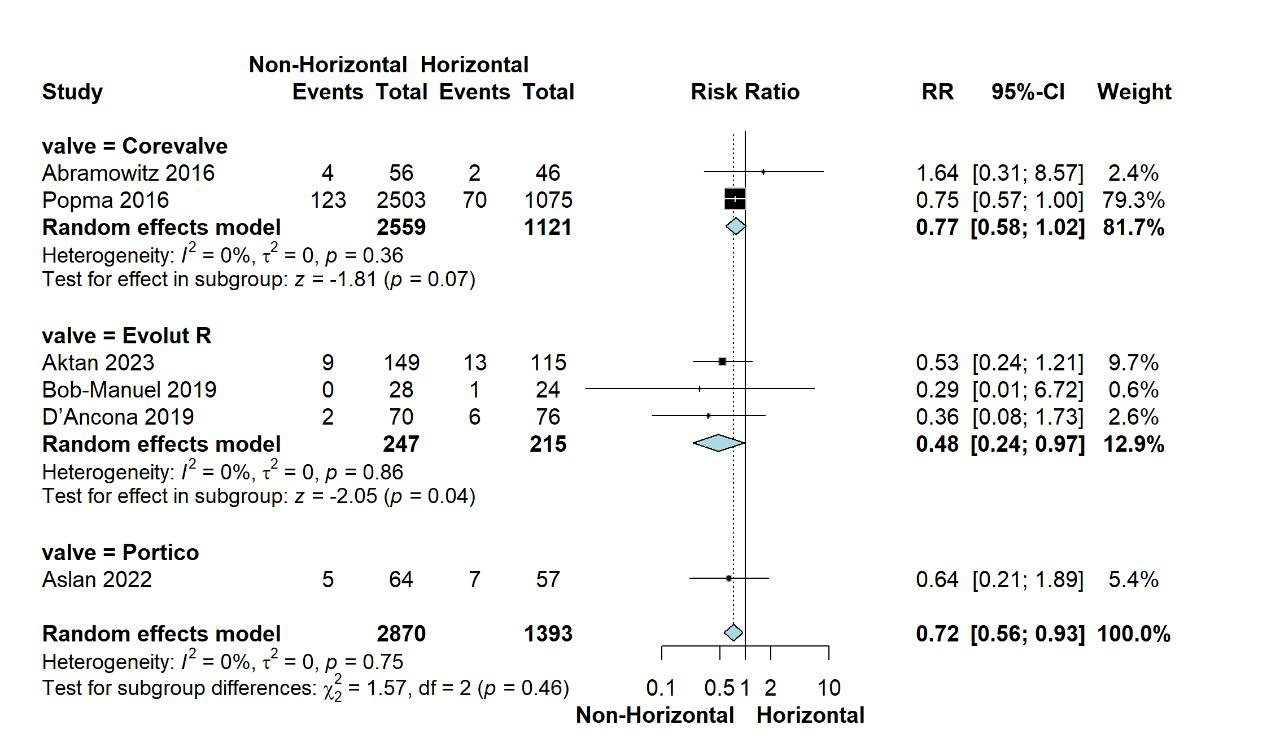


**Figure S1**


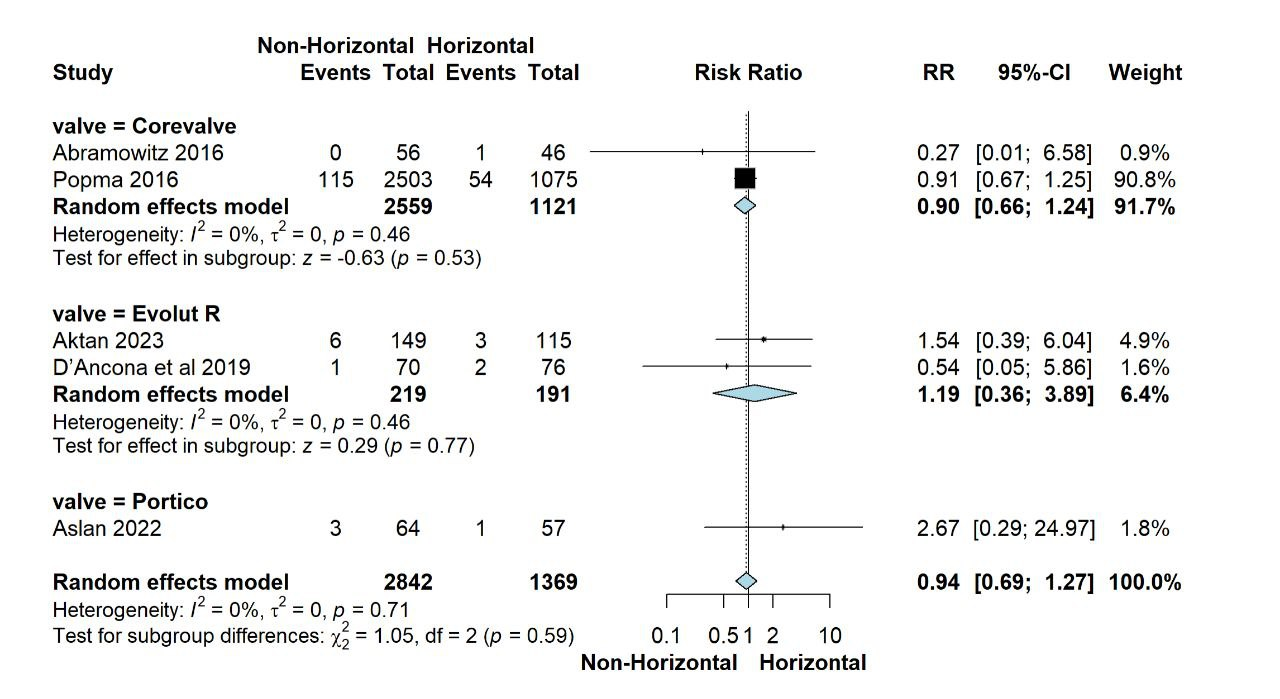


**Figure S2**

**
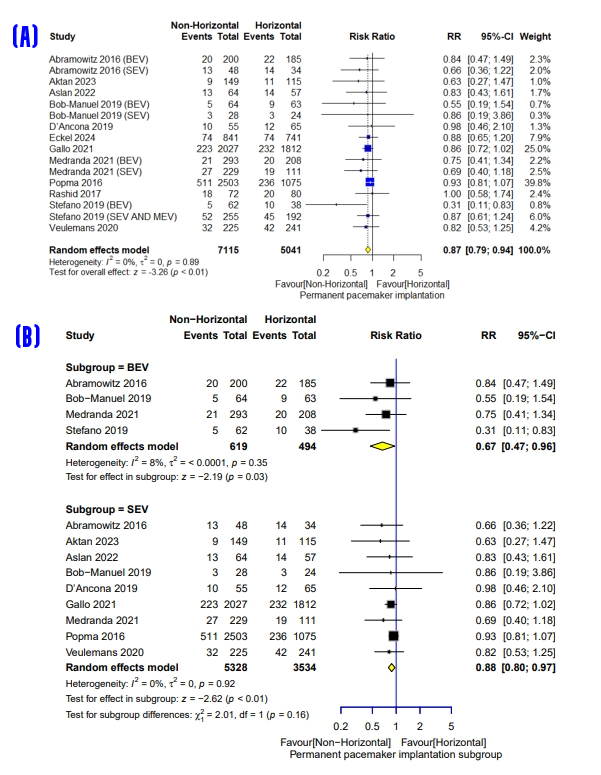
**

**Figure S3**


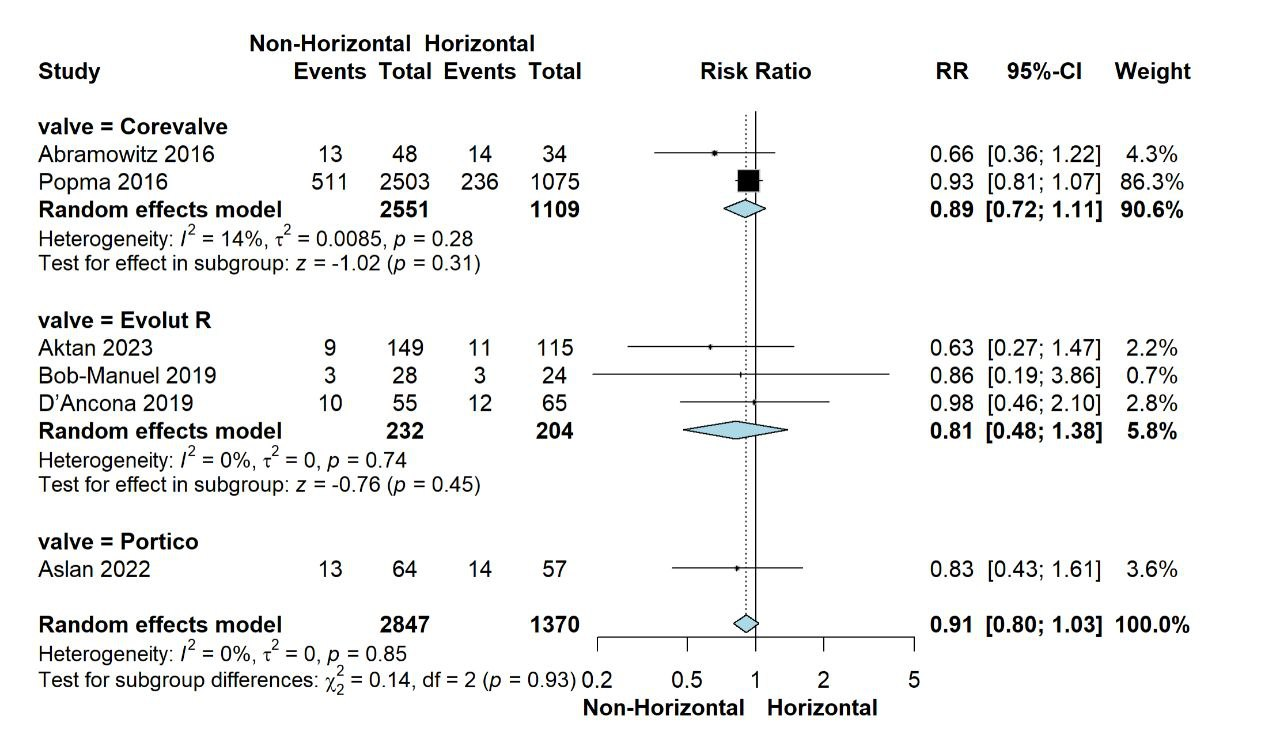


**Figure S4**

**
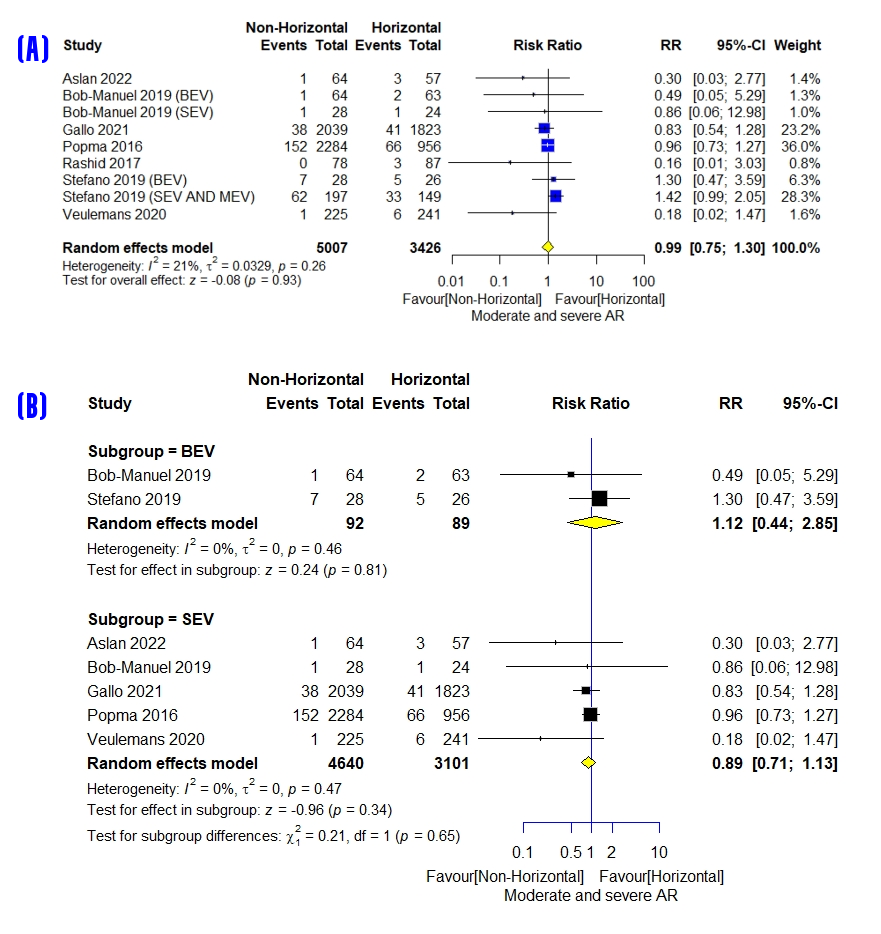
**

**Figure S5**

**
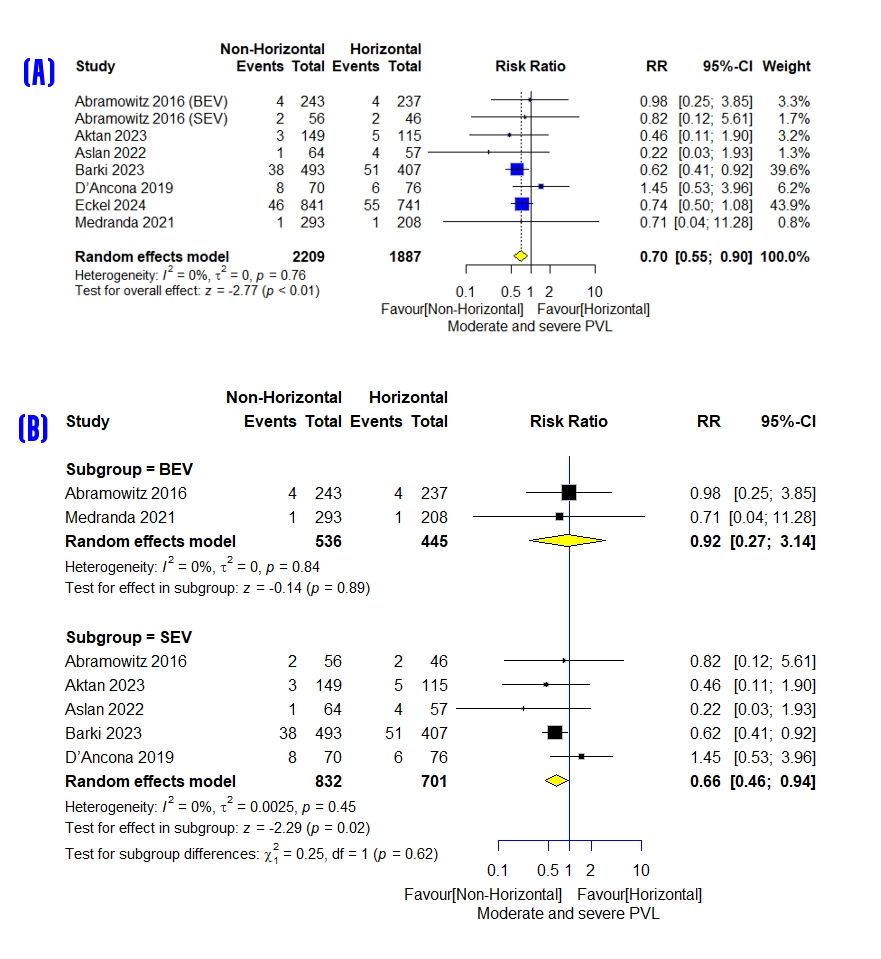
**

**Figure S6**

**
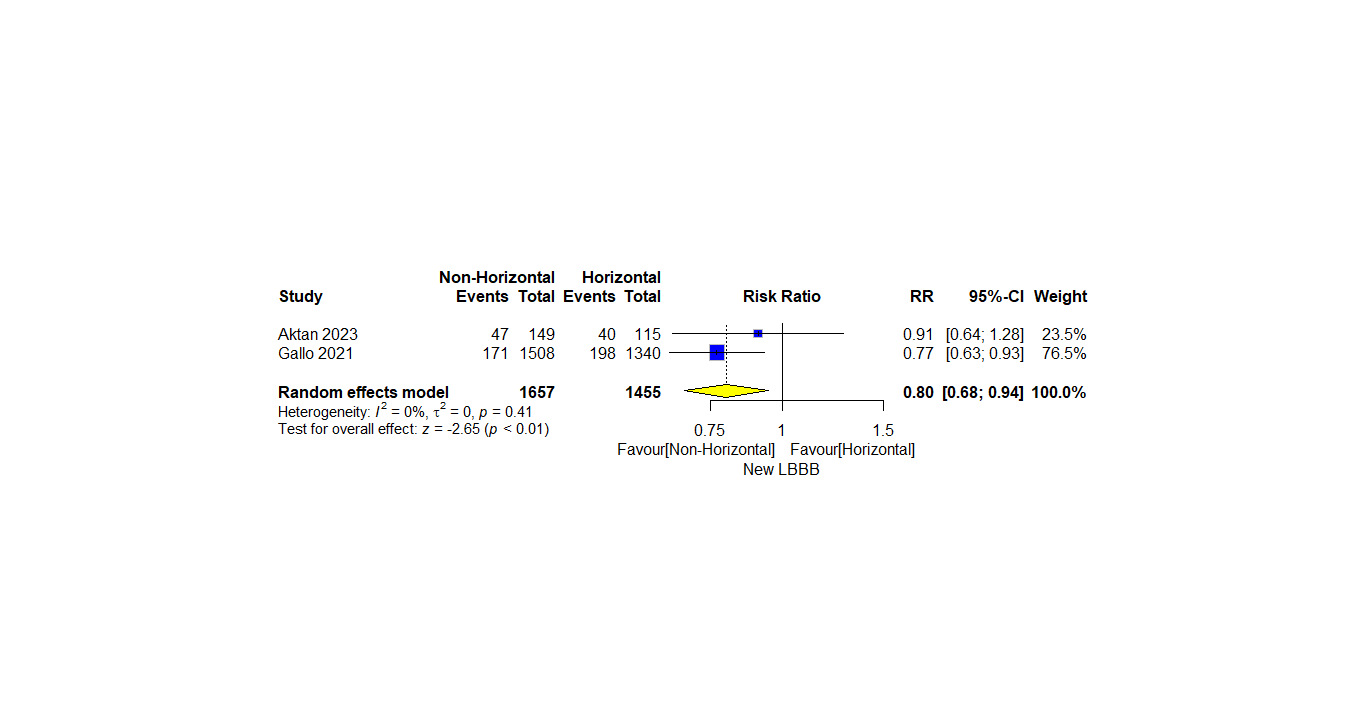
**

**Figure S7**

**
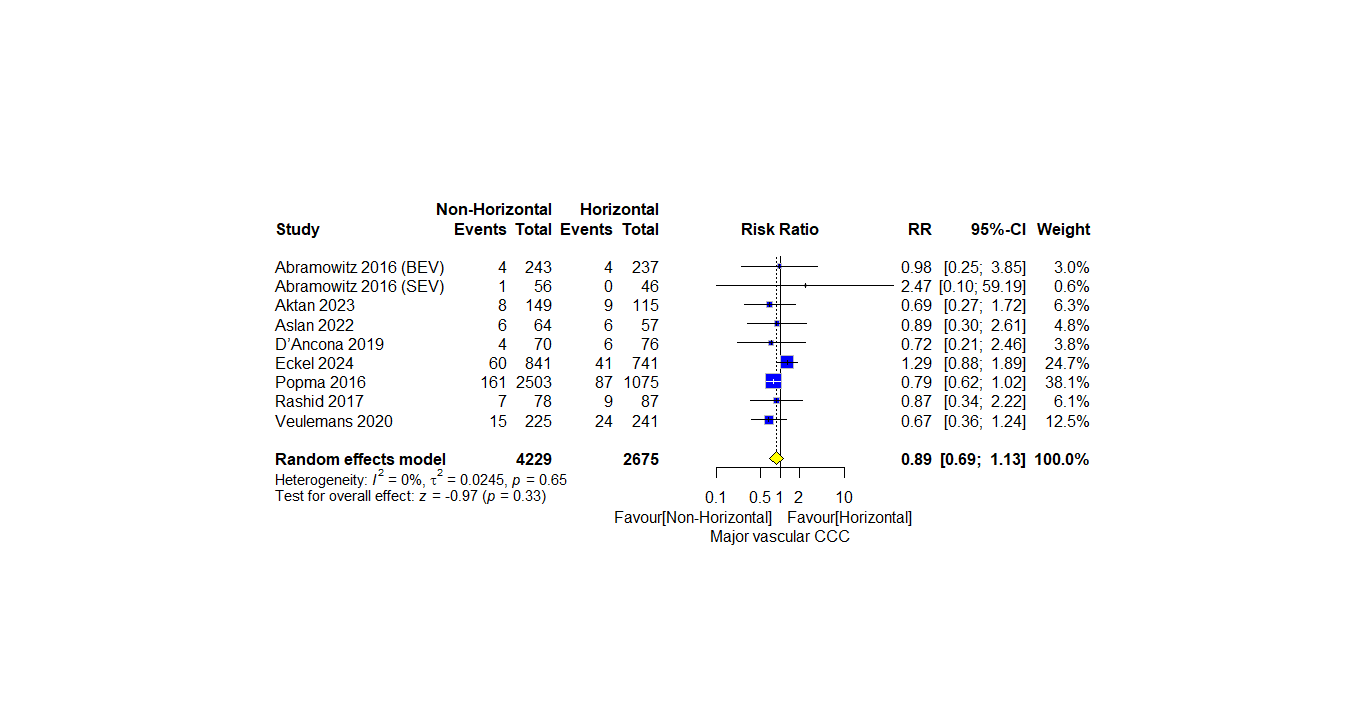
**

**Figure S8**


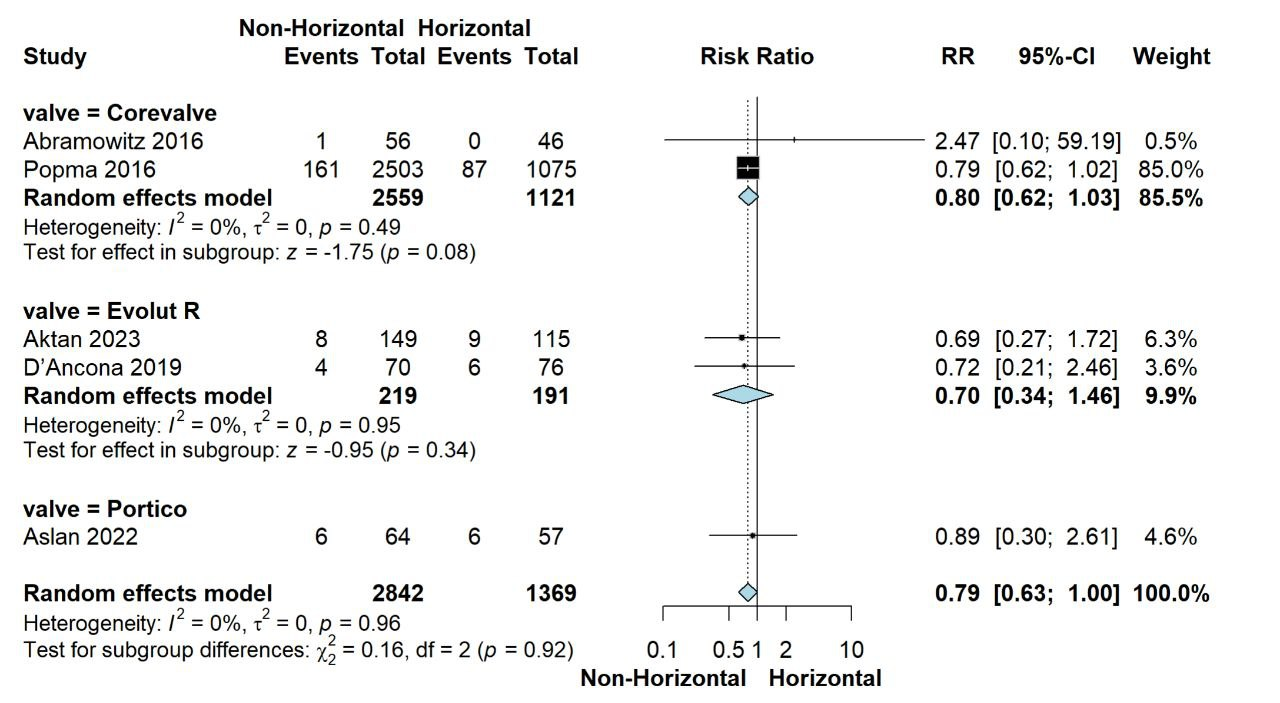


**Figure S9**

**
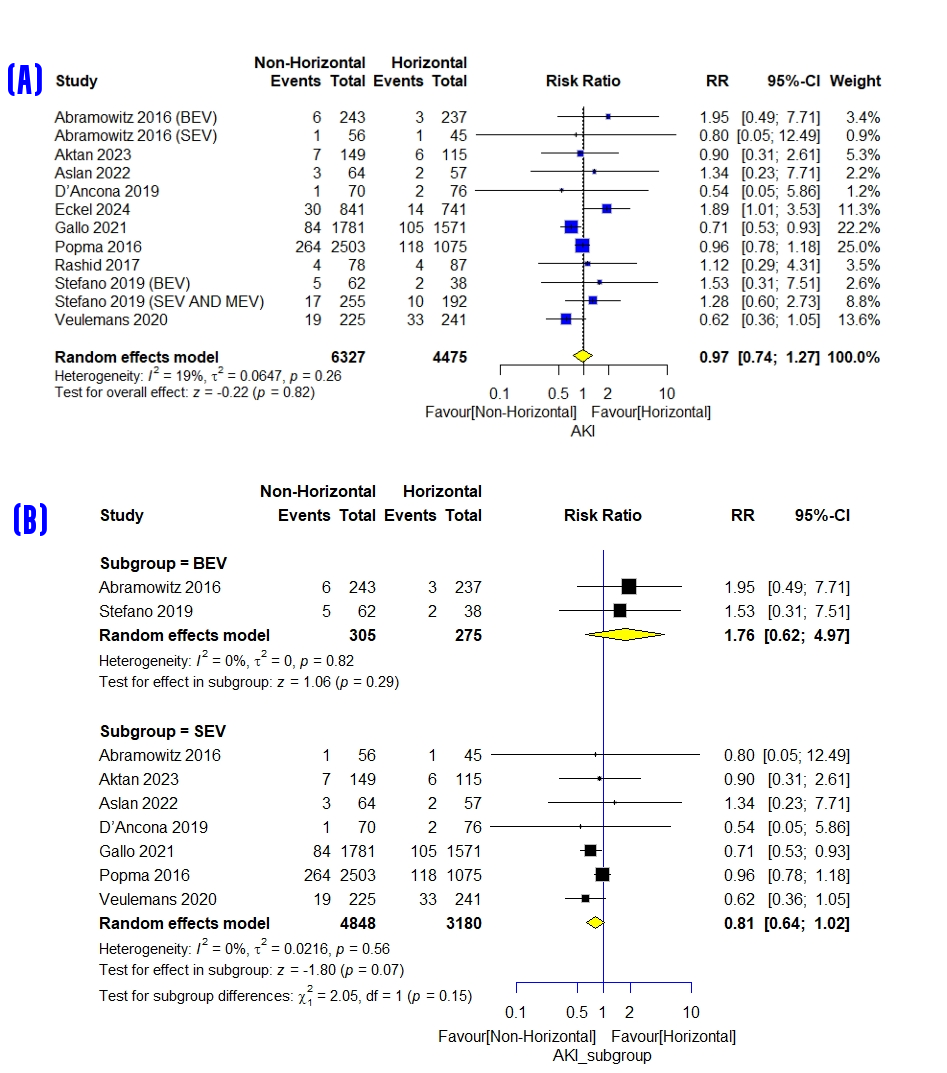
**

**Figure S10**


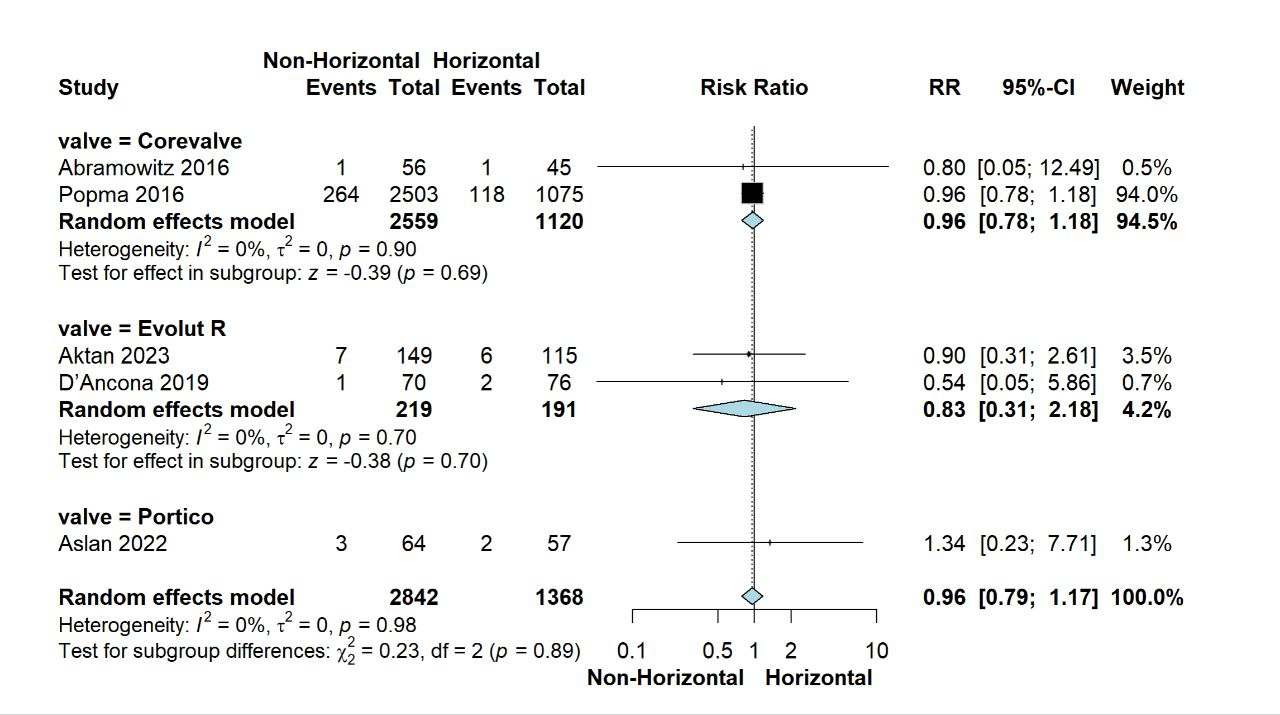


**Figure S11**

**
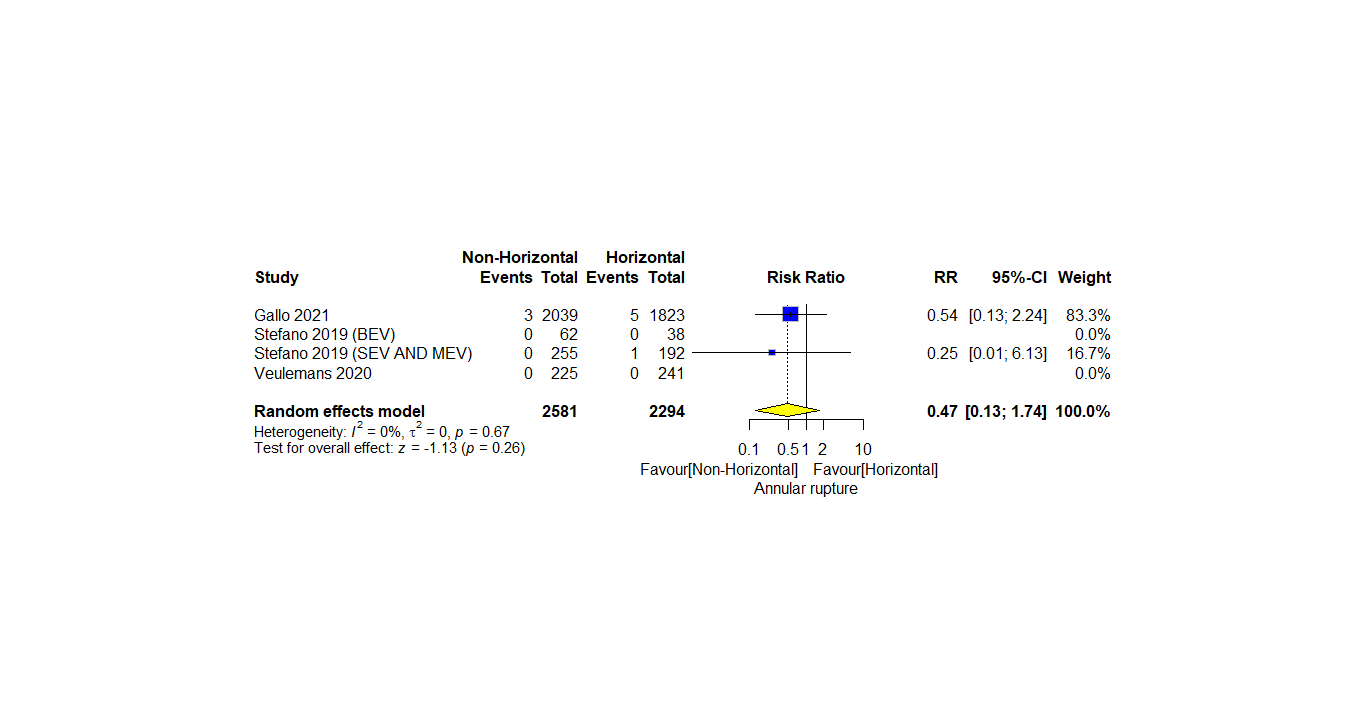
**

**Figure S12**

**
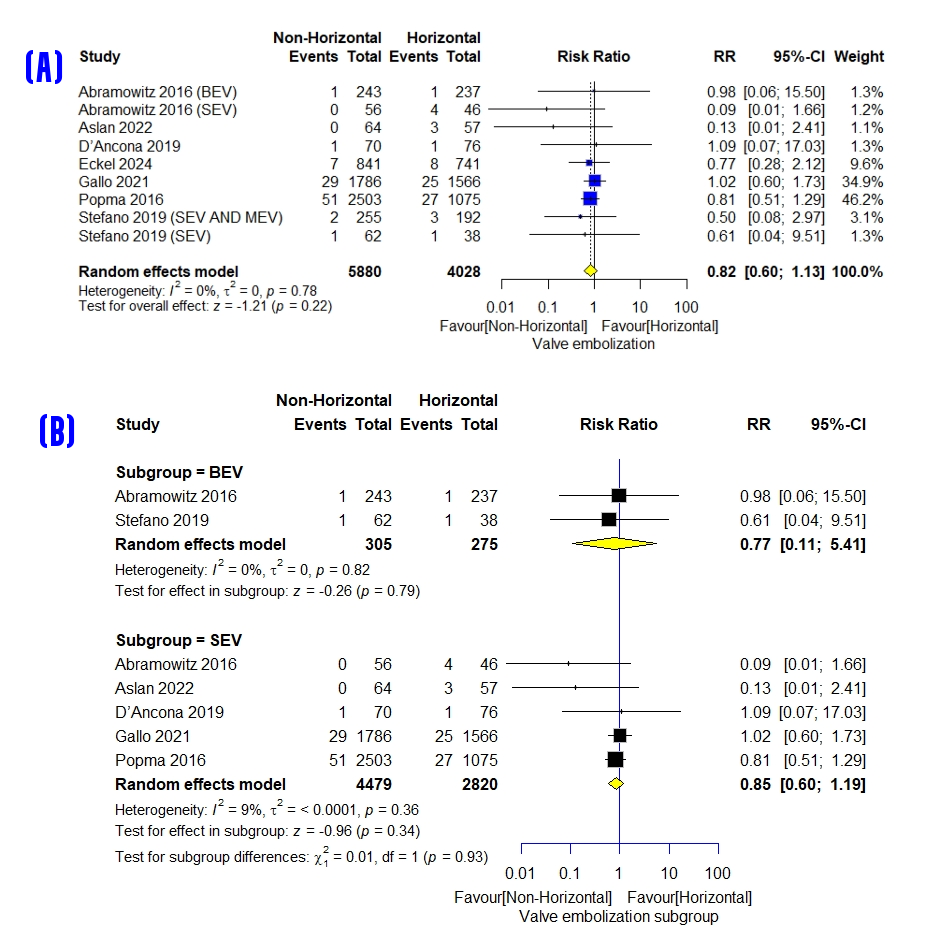
**

**Figure S13**

**
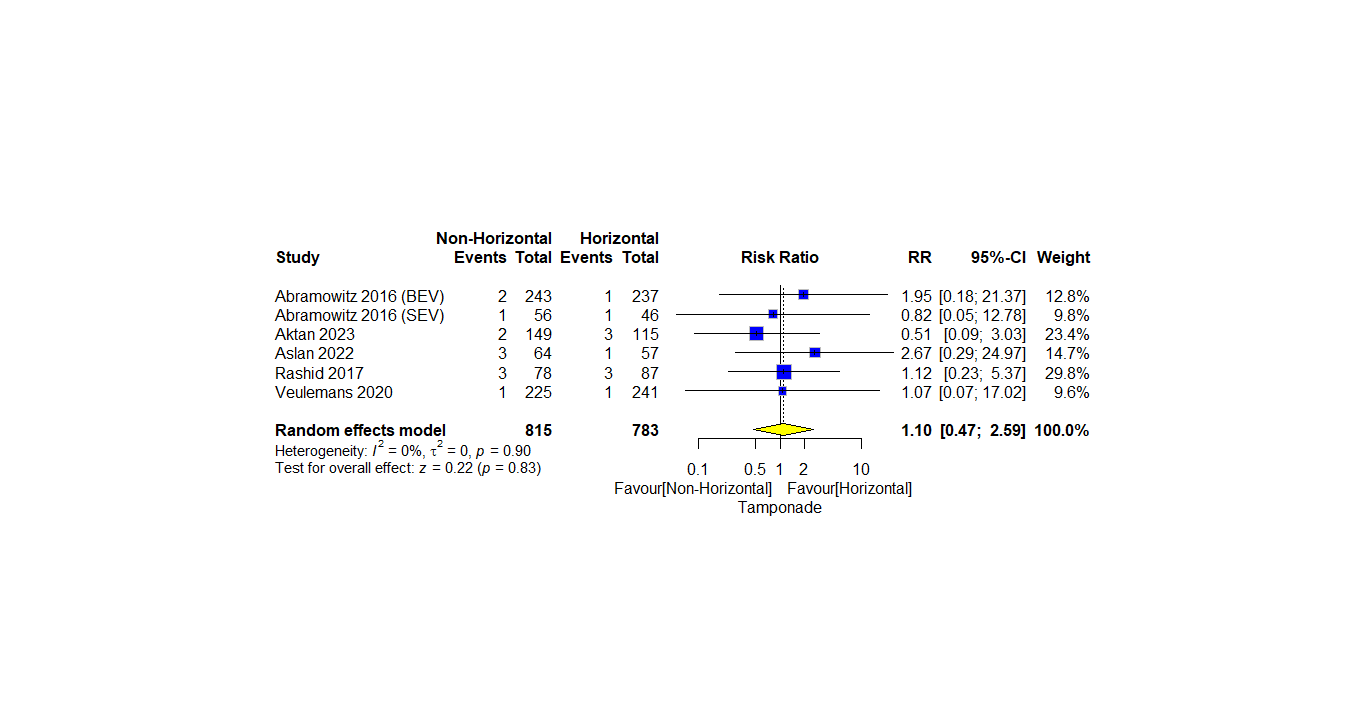
**

**Figure S14**

**
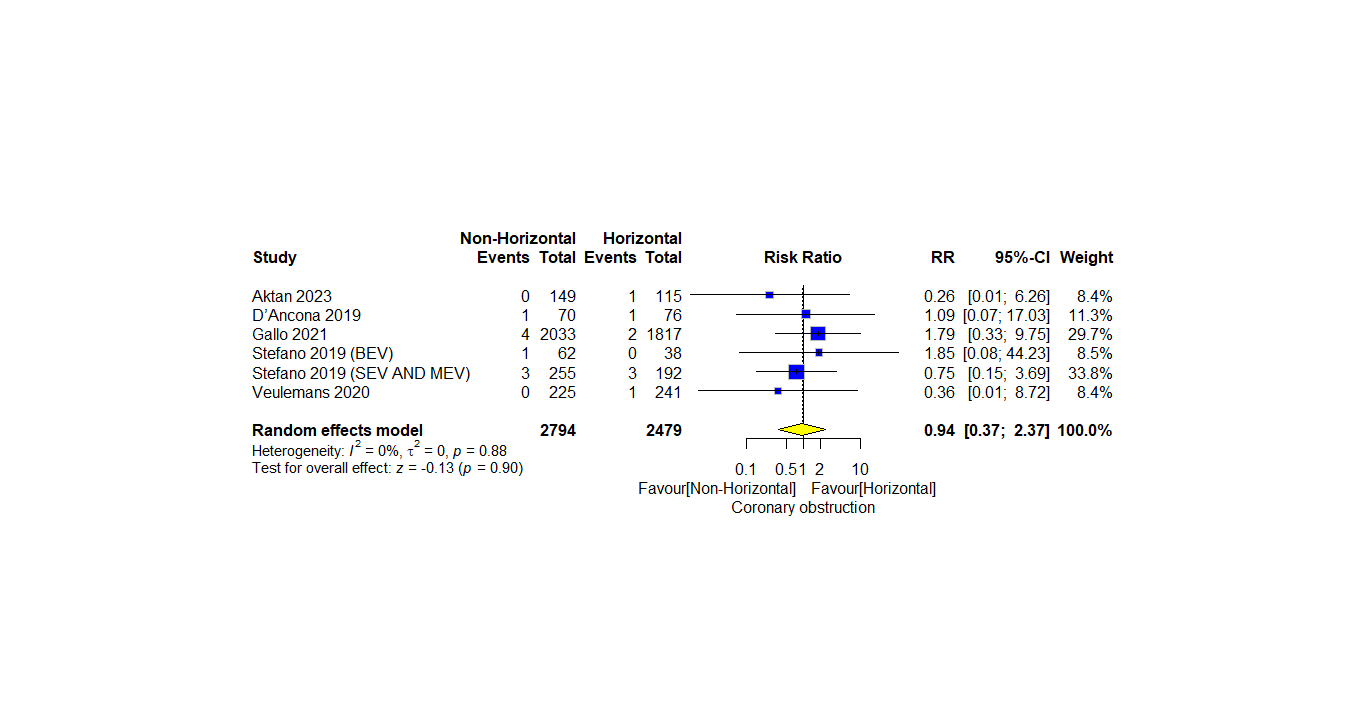
**

**Figure S15**

**
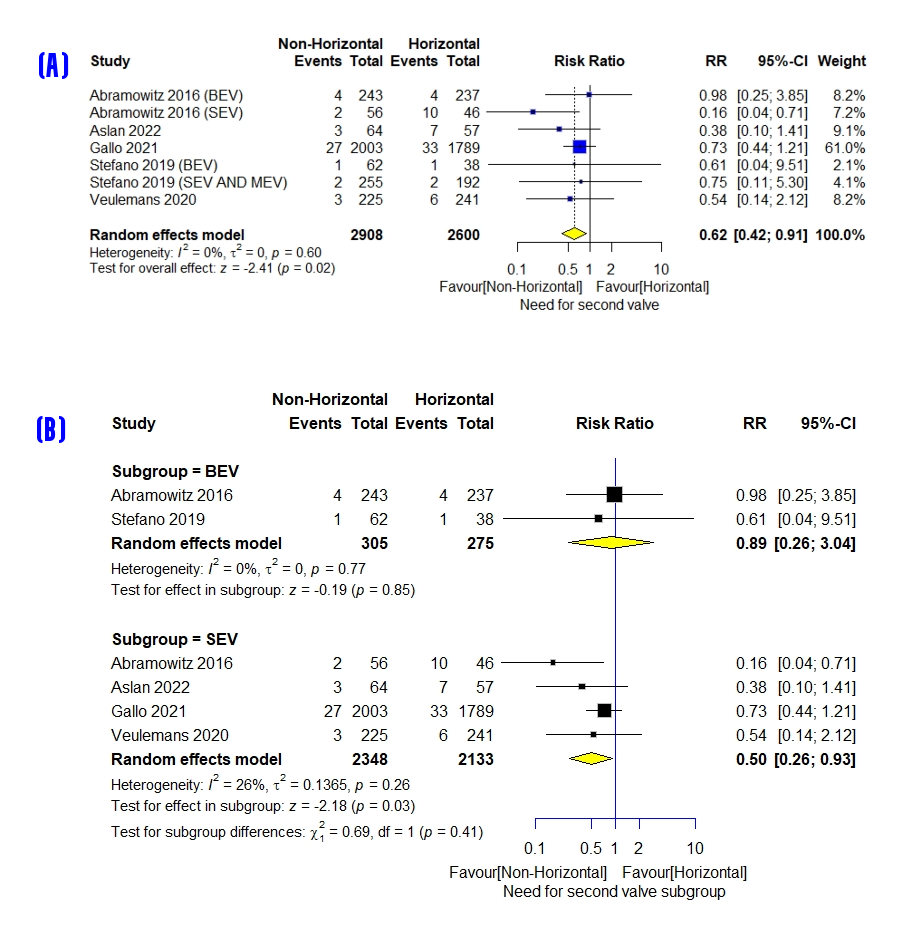
**

**Figure S16**

**
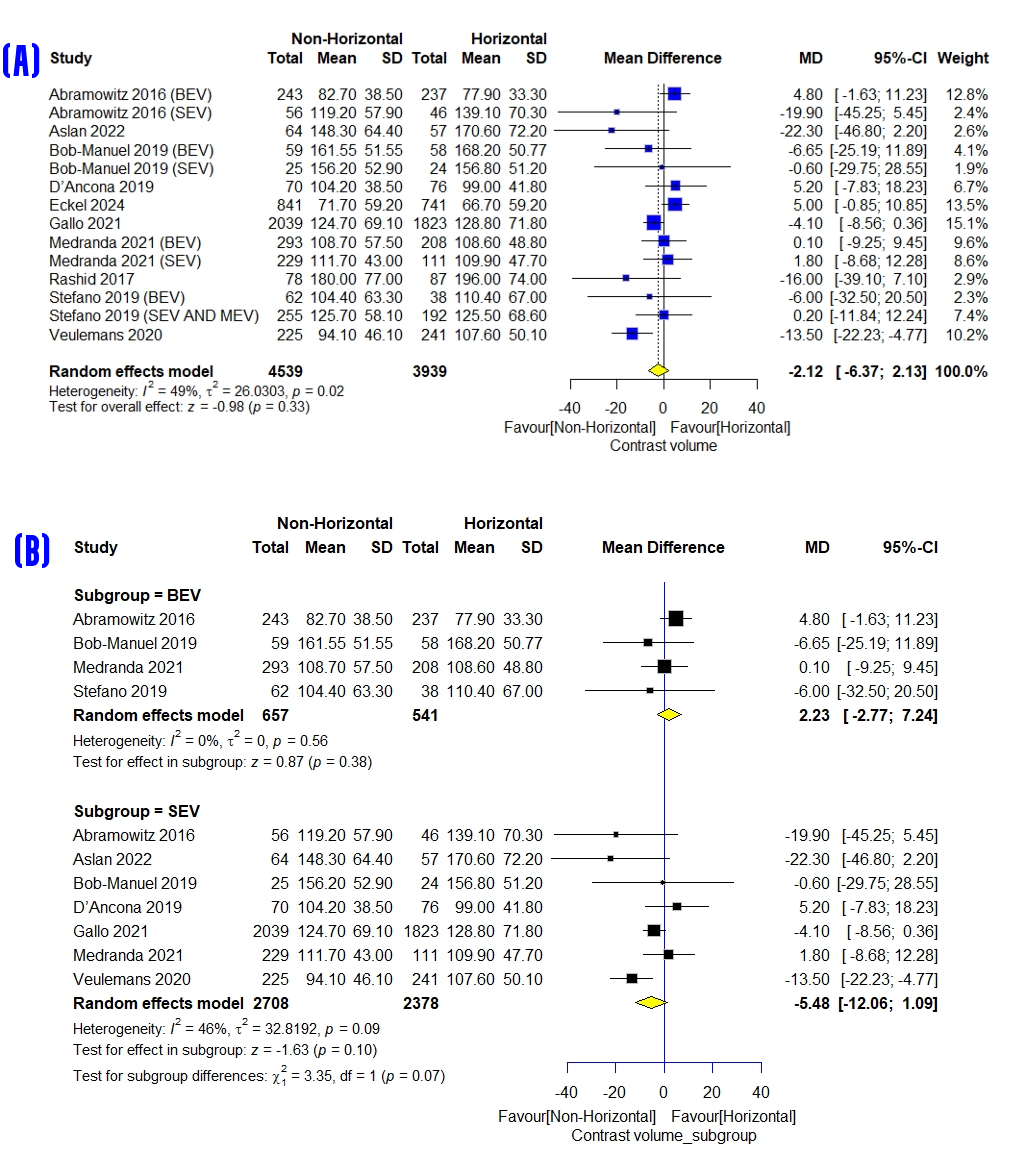
**

**Figure S17**

**
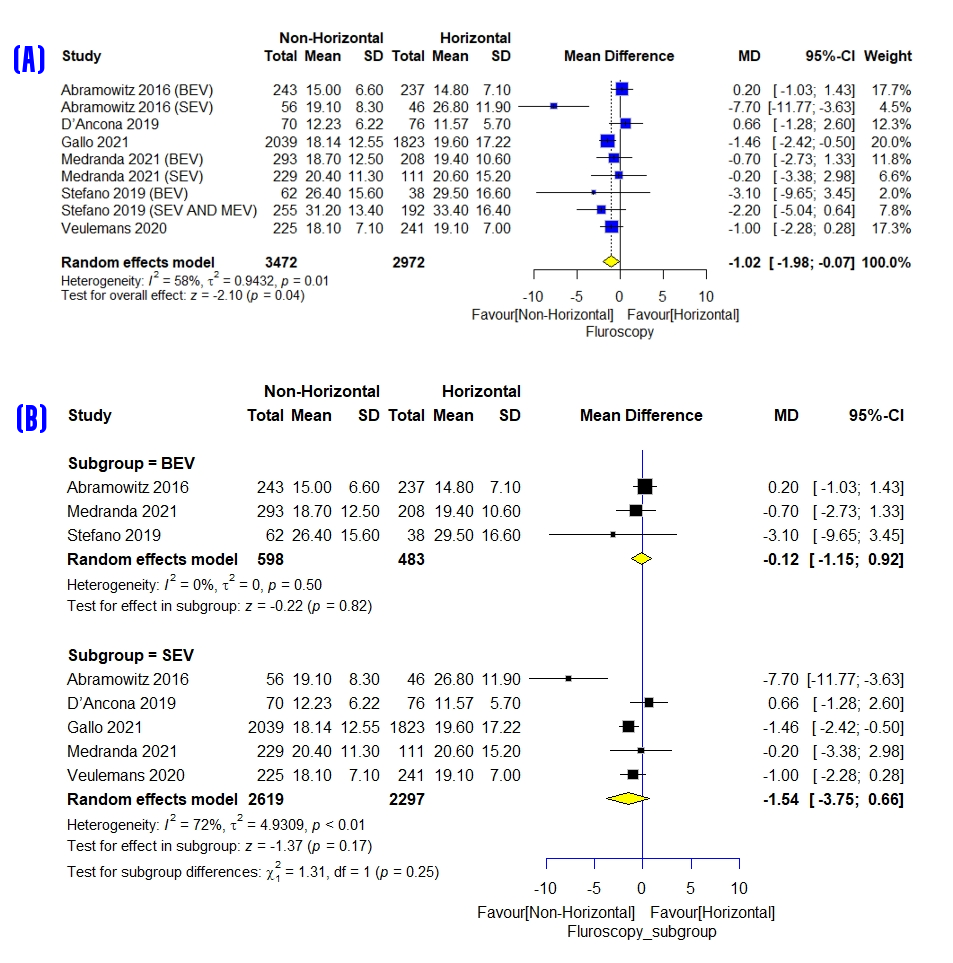
**

**Figure S18**

**
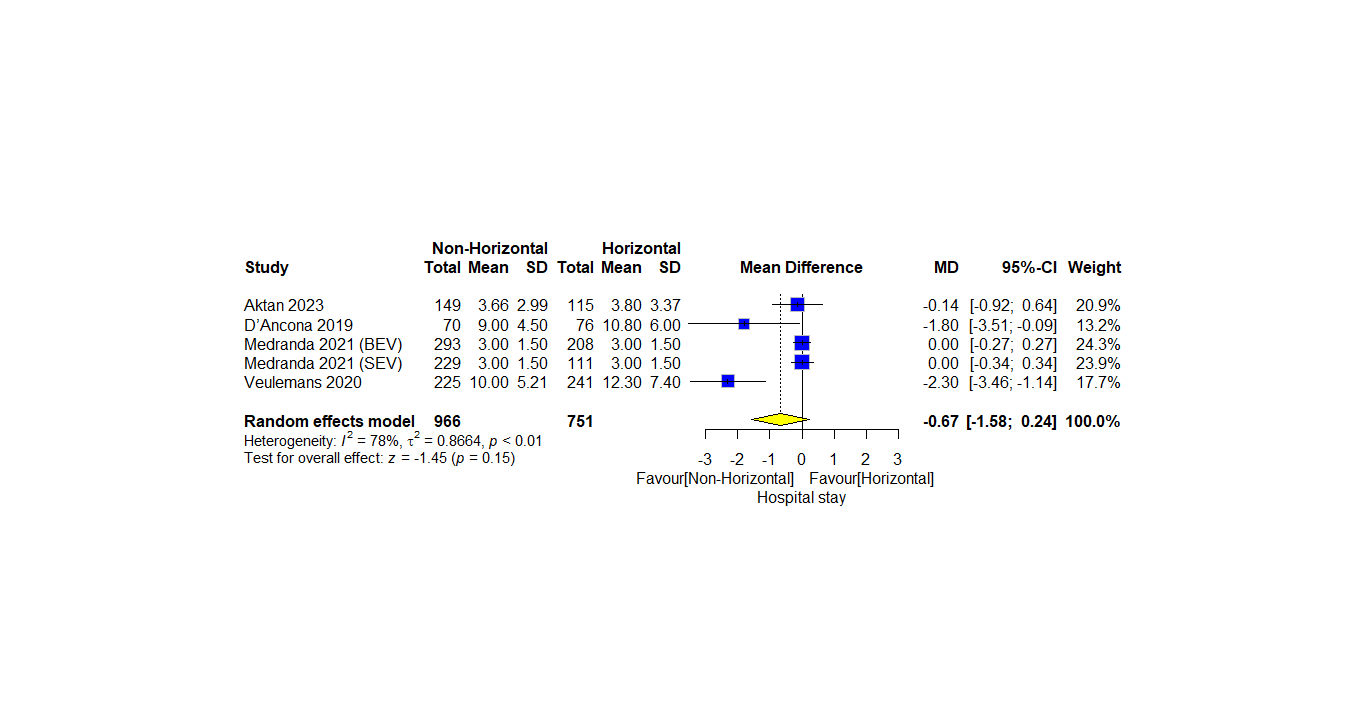
**

**Figure S19**
