# Supplementary material for: Impact of aortic angulation on outcomes in transcatheter aortic valve replacement with balloon-expandable and self-expanding valves: a systematic review and meta-analysis
Source: Cardiovasc Interv Ther. 2025 Jul 18;40(4):746–66. doi: 10.1007/s12928-025-01169-8 (PMC12431928; doi:10.1007/s12928-025-01169-8)
Supplement: Supplementary file 2 — Supplementary file2 (DOCX 26 KB) [file 12928_2025_1169_MOESM2_ESM.docx]

**Supplementary table 1: Procedure characteristics.**

| Study ID | Valve used | Femoral access | | subclavian access | | Pre-dilation | | Post-dilation | | valve type | | Valve size | | | | | | | |
| --- | --- | --- | --- | --- | --- | --- | --- | --- | --- | --- | --- | --- | --- | --- | --- | --- | --- | --- | --- |
|  |  |  |  |  |  |  |  |  |  |  |  | 23 mm | | 26 mm | | 29 mm | | 34 mm | |
|  |  | Non-HA | HA | Non-HA | HA | Non-HA | HA | Non-HA | HA | Non-HA | HA | Non-HA | HA | Non-HA | HA | Non-HA | HA | Non-HA | HA |
| Abramowitz et al 2016 | SEV | 55 (98.2) | 45 (97.8) | 1 (1.8) | 0 (0) | 9 (16.1) | 8 (17.4) | 8 (14.3) | 22 (47.8) | CoreValve | CoreValve | 4 (7.1) | 0 (0) | 15 (26.8) | 9 (19.6) | 27 (48.2) | 13 (28.3) | NA | NA |
| Abramowitz et al 2016 | BEV | 206 (84.8) | 201 (84.8) | 1 (0.4) | 4 (1.7) | 165 (67.9) | 150 (63.3) | 22 (9.1) | 22 (9.3) | Sapien/Sapien-XT/Sapien 3 | Sapien /Sapien-XT/Sapien 3 | 57 (23.5) | 70 (29.5) | 120 (49.4) | 98 (41.4) | 66 (27.2) | 69 (29.1) | 0 (0) | 0 (0) |
| Aktan et al 2023 | SEV | 149 (100) | 115 (100) | NA | NA | 38 (25.5) | 28 (24.6) | 35 (23.5) | 24 (21.1) | Evolut R | Evolut R | NA | NA | NA | NA | NA | NA | NA | NA |
| Aslan et al 2022 | SEV | 64 (100) | 57 (100) | NA | NA | 59 (92.2) | 50 (87.7) | 29 (45.3) | 30 (52.6) | portico | portico | 4 (6.2) | 2 (3.5) | NA | NA | 28 (43.8) | 25 (43.9) | NA | NA |
| Barki et al 2023 | SEV | NA | NA | NA | NA | NA | NA | NA | NA | Acurate neo and neo2 | Acurate neo and neo2 | NA | NA | NA | NA | NA | NA | NA | NA |
| Bob-Manuel et al 2019 | SEV | 25 (89.3) | 24 (100) | 2 (7.1) | 0 (0) | 1 (3.6) | 5 (19) | 3 (10.7) | 6 (25) | Evolut R | Evolut R | 1 (3.6) | 6 (25) | 12 (42.9) | 3 (12.5) | 15 (53.6) | 13 (54.2) | 0 (0) | 2 (8.3) |
| Bob-Manuel et al 2019 | BEV | 61 (95.3) | 61 (96.8) | 0 (0) | 1 (1.6) | 51 (79.7) | 55 (87.3) | 13 (20.3) | 12 (19) | Sapien 3/Sapien XT | Sapien 3 /Sapien XT | 18 (28.1) | 20 (31.7) | 27 (42.2) | 27 (42.9) | 16 (25) | 17 (27) | 1 (1.6) | 0 (0) |
| D’Ancona et al 2019 | SEV | NA | NA | NA | NA | 39 (55.7) | 28 (36.8) | 8 (11.4) | 11 (14.5) | Evolut-R | Evolut-R | 0 (0) | 6 (7.9) | 22 (31.4) | 13 (17.1) | 39 (55.7) | 43 (56.6) | 9 (12.9) | 14 (18.4) |
| Eckel et al 2024 | SEV and BEV | NA | NA | NA | NA | 567 (67.5) | 496 (67) | 184 (22.3) | 165 (22.5) | Sapien 3 Ultra and Acurate neo2 | Sapien 3 Ultra and Acurate neo2 | NA | NA | NA | NA | NA | NA | NA | NA |
| Gallo et al 2021 | SEV | 2039 (100) | 1823 (100) | NA | NA | NA | NA | NA | NA | Evolut-R/ Acurate neo | Evolut-R/ Acurate neo | NA | NA | NA | NA | NA | NA | AN | NA |
| Medranda et al 2021 | SEV | 229 (100) | 111 (100) | 0 (0) | (0) | NA | NA | NA | NA | Evolut PRO/PRO+ | Evolut PRO/PRO+ | NA | NA | NA | NA | NA | NA | NA | NA |
| Medranda et al 2021 | BEV | 293 (100) | 208 (100) | 0 (0) | 0 (0) | NA | NA | NA | NA | Sapien 3 | Sapien 3 | NA | NA | NA | NA | NA | NA | NA | NA |
| Popma et al 2016 | SEV | 1998 (79.8) | 896 (83.3) | 144 (5.8) | 49 (4.6) | 2080 (83.1) | 885 (82.3) | 592 (23.7) | 262 (24.4) | CoreValve | CoreValve | 45 (1.8) | 16 (1.5) | 606 (24.2) | 256 (23.8) | 1113 (44.5) | 459 (42.7) | NA | NA |
| Rashid et al 2017 | MEV | 78 (100) | 87 (100) | 0 (0) | 0 (0) | NA | NA | 2 (3) | 3 (3) | Lotus | Lotus | NA | NA | NA | NA | NA | NA | NA | NA |
| Stefano et al 2019 | BEV | 62 (100) | 38 (100) | 0 (0) | 0 (0) | 18 (29) | 12 (31.6) | 7 (11.3) | 4 (10.5) | SAPIEN 3 | SAPIEN 3 | 23 (37.1) | 13 (34.2) | 27 (43.5) | 13 (34.2) | 12 (19.4) | 12 (31.6) | 0 (0) | 0 (0) |
| Stefano et al 2019 | SEV and MEV | 255 (100) | 192 (100) | 0 (0) | 0 (0) | 187 (73.3) | 134 (69.8) | 66 (26) | 34 (17.7) | Evolut R/ Evolut PRO/Acurate neo/ Portico/ Directflow/ Lotus | Evolut R/ Evolut PRO/Acurate neo/ Portico/ Directflow/ Lotus | 36 (14.2) | 20 (10.4) | 22 (8.7) | 24 (12.5) | 66 (26) | 47 (24.5) | 13 (5.1) | 5 (2.6) |
| Veulemans et al 2020 | SEV | 225 (100) | 241 (100) | 0 (0) | (0) | 102 (55.3) | 113 (46.9) | 23 (10.2) | 31 (12.9) | Evolut R/CoreValve EvolutPRO | Evolut R/CoreValve EvolutPRO | 7 (3.1) | 0 (0) | 85 (37.8) | 67 (27.8) | 90 (40) | 110 (45.6) | 43 (19.1) | 64 (26.6) |

**SEV:** self-expandable valves**; BEV:** ballon-expandable valves**; MEV:** mechanically-expandable valve**; HA:** horizontal aorta.
