# Supplementary material for: Impact of aortic angulation on outcomes in transcatheter aortic valve replacement with balloon-expandable and self-expanding valves: a systematic review and meta-analysis
Source: Cardiovasc Interv Ther. 2025 Jul 18;40(4):746–66. doi: 10.1007/s12928-025-01169-8 (PMC12431928; doi:10.1007/s12928-025-01169-8)
Supplement: Supplementary file 3 — Supplementary file3 (DOCX 17 KB) [file 12928_2025_1169_MOESM3_ESM.docx]

**Supplementary Table 2: Risk of bias assessment with the new-castle Ottawa scale (NOS).**

| Study ID | Valve group | Selection | | | | Comparability | Outcome | | | Overall Score | quality |
| --- | --- | --- | --- | --- | --- | --- | --- | --- | --- | --- | --- |
|  |  | Representativeness of the exposed cohort | Selection of the non-exposed cohort | Ascertainment of exposure | Demonstration that outcome of interest was not present at start of study | Comparability of cohorts on the basis of the design or analysis | Assessment of outcome | Was follow-up long enough for outcomes to occur | Adequacy of follow up of cohorts |  |  |
| Abramowitz et al 2016 | SEV | * | * | * | * | * | * | * | * | 8 | Good |
| Abramowitz et al 2016 | BEV | * | * | * | * | * | * | * | * | 8 | Good |
| Aktan et al 2023 | SEV | * | * | * | * | * | * | * | * | 8 | Good |
| Aslan et al 2022 | SEV | * | * | * | * | * | * | * | * | 8 | Good |
| Barki et al 2023 | SEV | * | * | * | * | 0 | * | * | * | 7 | Good |
| Bob-Manuel et al 2019 | SEV | * | * | * | * | * | * | * | * | 8 | Good |
| Bob-Manuel et al 2019 | BEV | * | * | * | * | * | * | * | * | 8 | Good |
| D’Ancona et al 2019 | SEV | * | * | * | * | * | * | * | * | 8 | Good |
| Eckel et al 2024 | SEV and BEV | * | * | * | * | 0 | * | * | * | 7 | Good |
| Gallo et al 2021 | SEV | * | * | * | * | * | * | * | * | 8 | Good |
| Medranda et al 2021 | SEV | * | * | * | * | * | _ | * | * | 7 | Good |
| Medranda et al 2021 | BEV | * | * | * | * | * | * | * | * | 8 | Good |
| Popma et al 2016 | SEV | * | * | * | * | * | * | * | * | 8 | Good |
| Rashid et al 2017 | MEV | * | * | * | * | * | * | * | * | 8 | Good |
| Stefano et al 2019 | BEV | * | * | * | * | * | * | * | * | 8 | Good |
| Stefano et al 2019 | SEV and MEV | * | * | * | * | 0 | * | * | * | 7 | Good |
| Veulemans et al 2020 | SEV | * | * | * | * | * | 0 | * | * | 7 | Good |
