# Supplementary material for: Impact of aortic angulation on outcomes in transcatheter aortic valve replacement with balloon-expandable and self-expanding valves: a systematic review and meta-analysis
Source: Cardiovasc Interv Ther. 2025 Jul 18;40(4):746–66. doi: 10.1007/s12928-025-01169-8 (PMC12431928; doi:10.1007/s12928-025-01169-8)
Supplement: Supplementary file 4 — Supplementary file4 (DOCX 16 KB) [file 12928_2025_1169_MOESM4_ESM.docx]

**Supplementary Table 3: Definitions of outcomes according to VARC criteria.**

| Study ID | VARC criteria |
| --- | --- |
| Abramowitz et al 2016 | VARC-2^(1)^ |
| Aktan et al 2023 | VARC-3^(2)^ |
| Aslan et al 2022 | VARC-3^(2)^ |
| Barki et al 2023 | VARC-3^(2)^ |
| Bob-Manuel et al 2019 | VARC-2^(1)^ |
| D’Ancona et al 2019 | VARC-2^(1)^ |
| Eckel et al 2024 | VARC-3^(2)^ |
| Gallo et al 2021 | VARC-2^(1)^ |
| Medranda et al 2021 | VARC-2^(1)^ |
| Popma et al 2016 | VARC-2^(1)^ |
| Rashid et al 2017 | VARC-2^(1)^ |
| Stefano et al 2019 | VARC-2^(1)^ |
| Veulemans et al 2020 | VARC-2^(1)^ |

**Abbreviation: VARC;** Valve Academic Research Consortium.

**References:**

1. Kappetein AP, Head SJ, Généreux P, Piazza N, Van Mieghem NM, Blackstone EH, et al. Updated standardized endpoint definitions for transcatheter aortic valve Implantation: The valve academic research consortium-2 consensus document (varc-2). Eur J Cardio-Thoracic Surg. (2012) 42:S45–60. Doi: 10.1093/ejcts/ezs533
2. Varc-3 Writing C, Genereux P, Piazza N, et al. Valve Academic Research Consortium-3: Updated Endpoint Definitions for Aortic Valve Clinical Research. J Am Coll Cardiol. 2021; 77:2717–2746.

**Supplementary Table 4: Definitions of the aortic angle**

| **Abramowitz 2016** | **AA was defined as the angle between the horizontal plane and the plane of the aortic annulus.** |
| --- | --- |
| **Aktan 2023** | **AA defined as the angle between the horizontal plane and the plane of the aortic annulus.** |
| **Aslan 2022** | **AA defined as the angle between the plane of the aortic annulus and the horizontal plane.** |
| **Barki 2023** | **AA defined as the angle between the horizontal plane and the plane of the aortic annulus in the coronal projection larger than or equal to 48 degrees,** |
| **Bob-Manuel 2019** | **AA defined as the angle between the horizontal plane and the plane of the aortic annulus** |
| **D’Ancona 2019** | **AA was defined as the angle between the AV annulus plane and the horizontal plane** |
| **Eckel 2024** | **AA is measured as the angle between the virtual basal ring of the aortic annulus and the horizontal** |
| **Gallo 2021** | **The AA was defined as the angle between the virtual basal ring and the horizontal plane in a coronal projection.** |
| **Medranda 2021** | **AA was calculated from a coronal projection at the level of the aortic annulus and was defined as the angle between the horizontal plane and the plane of the aortic annulus.** |
| **Popma 2016** | **AoV angulation was determined by angle of the aortic annulus and the horizontal plane in the end-systolic coronal view.** |
| **Rashid 2017** | **AA defined as the angle between the aortic annulus plane and the horizontal plane in a coronal projection** |
| **Stefano 2019** | **AA was defined as the angle between the horizontal plane and the plane of the aortic annulus,**  **calculated from a coronal projection at the level of the aortic annulus** |
| **Veulemans 2020** | **The ARA was defined as the angle between the horizontal plane and the plane of the aortic annulus, calculated from a coronal projection.** |
